# Pancreatic stellate cells have adipogenic and fibrogenic potentials but only show increased pro-fibrogenic propensity upon aging

**DOI:** 10.1016/j.redox.2025.103791

**Published:** 2025-07-29

**Authors:** George A. Soultoukis, Marina Leer, Richard Kehm, Laura Villacorta, Vladimir Benes, Tilman Grune, Annika Höhn, Tim J. Schulz

**Affiliations:** a Department of Adipocyte Development and Nutrition, German Institute of Human Nutrition Potsdam-Rehbrücke (DIfE), Nuthetal, Germany; b German Center for Diabetes Research (DZD), München-Neuherberg, Germany; c Department of Molecular Toxicology, German Institute of Human Nutrition Potsdam-Rehbrücke, Nuthetal, Germany; d Genomics Core Facility, European Molecular Biology Laboratory (EMBL), Heidelberg, Germany; e Institute of Nutritional Science, University of Potsdam, Nuthetal, Germany

**Keywords:** Stellate cells, Mesenchymal cells, Fibrosis, Ectopic fat, Pancreatic cancer, Diabetes

## Abstract

Steatosis and fibrosis are two key pathological features of the aging pancreas that contribute to inflammation, metabolic dysfunction and degenerative changes of the tissue. Pancreatic stellate cells (SCs) are thought to be the main cellular source of ectopic adipocytes and fibrotic structures. SCs are fibroblast-like mesenchymal cells that reside in various microanatomies of the tissue and whose task under healthy, homeostatic conditions is to generate extracellular matrix (ECM) and maintenance signals to ensure tissue integrity and function. To examine pathological changes of the aging pancreas, we conducted single cell RNA-sequencing to analyze murine pancreatic cell heterogeneity and examined morphological changes of the aging exocrine pancreas, comparing young adult and aged wildtype mice. We specifically focused our analyses on SCs and their cellular interaction partners and mechanisms of paracrine crosstalk. Age-dependent transcriptional differences in these cell types occur on the level of ECM-related and pro-fibrotic expression signatures mediated through transforming growth factor (TGF) and platelet-derived growth factor (PDGF) signaling pathways. SCs can be divided into distinct subsets of activated (aSCs) and quiescent stellate cells (qSCs), based on distinct expression signatures and transcript levels of *Pdgfra* and *Pdgfrb*, which encode for the PDGF receptor alpha and -beta paralogs. Activated SCs, which display PDGF receptor alpha (PDGFRα) on their surface, exhibited subsets that appeared to be either pro-adipogenic or pro-fibrogenic at the transcriptomic level and aging promoted a pro-fibrogenic switch in aSCs. We conclude that pancreatic SCs are contributors to the age-related decline of the exocrine pancreas through an increased contribution of a pro-fibrotic microenvironment.

## Graphical abstract

**Figure:**
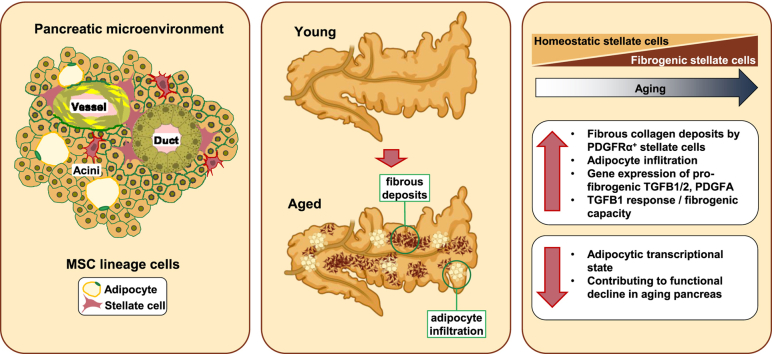

## 1 Introduction

The functional decline of the pancreas during aging is thought to exacerbate the onset of metabolic diseases, such as type-2 diabetes mellitus (T2DM), and digestive disorders in aged individuals [1,2]. Pancreatic tissue is organized into two distinct compartments with either exocrine or endocrine functions. The exocrine pancreas, which comprises more than 95 % of pancreatic mass, produces and subsequently releases digestive enzymes into the intestinal tract. It consists of blood vessels and the specialized acinar tissue composed of individual acini combined within lobules that are enveloped by fibrous capsules [3,4]. Pancreatic lobules consist of acinar cells that coalesce around ductal cells, which form the ducts that accommodate the flow of digestive enzymes towards the duodenum, thereby producing a key component of digestion [3]. The endocrine pancreas accounts for less than 3 % of pancreatic mass, and its main structure, the islets of Langerhans, is composed of a number of highly specialized endocrine cells and features a high degree of vascularization. Pancreatic islets secrete prominent hormones like glucagon and insulin, from α-cells and β-cells respectively, among others, thereby regulating systemic metabolism and glucose homeostasis [4].

Fatty infiltration, or steatosis, and accumulation of fibrosis are two hallmarks of a dysfunctional pancreas, resulting in impaired exocrine and endocrine functions and ultimately in the development of pancreatic cancer. Accordingly, age is the strongest predictor of fatty pancreas and has been associated with pancreatic cancer and poor prognosis [5,6]. Adipocyte accumulation in the pancreas has also been described as an independent risk factor for the development of T2DM [7]. Fat storage is observed in distinct regions, including acinar areas and between lobuli, i.e. interlobular, and is linked to chronic inflammatory processes and acute pancreatitis [8]. As a result of the persistent pro-inflammatory stimulus in an aging microenvironment, pancreatic fibrosis is thought to develop gradually, and is potentially linked to fatty infiltration as a cause of inflammation [8]. On the cellular level, activation of pancreatic stellate cells (SCs) results in excessive expression of type-1 collagen and fibrous deposits during aging, which in turn promotes exocrine and endocrine pancreatic tissue deterioration [9,10]. SCs also synthesize pro-inflammatory and pro-fibrotic factors, such as activin 1, and interleukin (IL)-1, platelet-derived growth factor (PDGF), and transforming growth factor-beta (TGFB), representing a source of signaling molecules to exacerbate inflammatory processes [9].

Alongside SCs, pancreatic endothelial cells (pECs) constitute a second pancreas-resident cell population which plays at least an auxiliary role in pancreatic inflammation and fibrosis [9,11,12]. pECs themselves secrete factors, such as Tumor necrosis factor-alpha (TNFA), TGFB1 and activin 1, that mediate a crosstalk with SCs and also contribute extracellular matrix (ECM) components including several collagens to the local microenvironment [[12], [13], [14]]. Under pathological conditions, functions of the endothelium can be compromised, resulting in immunocytes traversing the vascular endothelial cell layers of micro-vessels [15,16]. These invading immunocytes can infiltrate the pancreatic tissue causing inflammation and insulitis, which chronically can cause or aggravate fibrosis and pancreatitis, diabetes, and pancreatic cancer [12,15,17,18]. However, the precise contributions of each of the two cell populations, SCs or ECs, towards inflammation-linked pancreatic pathologies during aging are not fully characterized. Within the context of pancreatic fibrosis and adipogenesis, cell-to-cell interactions need to be further characterized to address the question of how pancreatic function is orchestrated in response to metabolic stimuli and disease-associated processes.

To examine the contribution of distinct cell types to the pathological changes of the aging pancreas, we conducted scRNA-seq to analyze murine pancreatic cell populations, specifically focusing on SCs and their paracrine crosstalk with other pancreatic cell subsets within the bulk of pancreatic tissue substance, the exocrine areas of the organ. These data together with morphological analyses revealed that aging leads to notable transcriptional changes in ECM-related and pro-fibrotic gene expression mediated by TGFB- and PDGF-signaling in SCs, and specifically drives a pro-fibrogenic switch in activated SC subsets. We conclude that a complex interplay between pancreatic stellate and endothelial cells, involving pro-inflammatory and pro-fibrogenic processes, might be key in modulating the onset of pancreatic pathology with age.

## 2 Results

### 2.1 scRNA-seq of heterogenous pancreatic cells uncovers distinct subsets of pancreatic stellate and endothelial cells

Pancreatic SCs reside in a complex microenvironment of exocrine and endocrine tissue components. To examine their interaction with distinct cell populations of the pancreas and their contribution to age-related changes of the tissue, we conducted scRNA-seq to assess cellular heterogeneity and age-related changes of defined cellular subsets at single cell resolution. Due to low relative abundance of endocrine cells (<3 %) in relation to exocrine cells (>90 %) [4], we aimed to capture both exocrine as well as endocrine cellular subsets for a better characterization of pathological cell-cell interactions including pro-fibrotic and pro-adipogenic cross-talks. We therefore initially utilized partially islet-enriched pancreas samples collected from adult (5-months-old) and from very aged (25-months-old) male C57Bl/6J mice. Cell suspensions were analyzed by droplet-based scRNA-seq, resulting in a post-QC-filtering total of 1680 cells, representing 932 cells from 5-months old and 748 cells from 25-month-old mice.

**Fig. 1:**
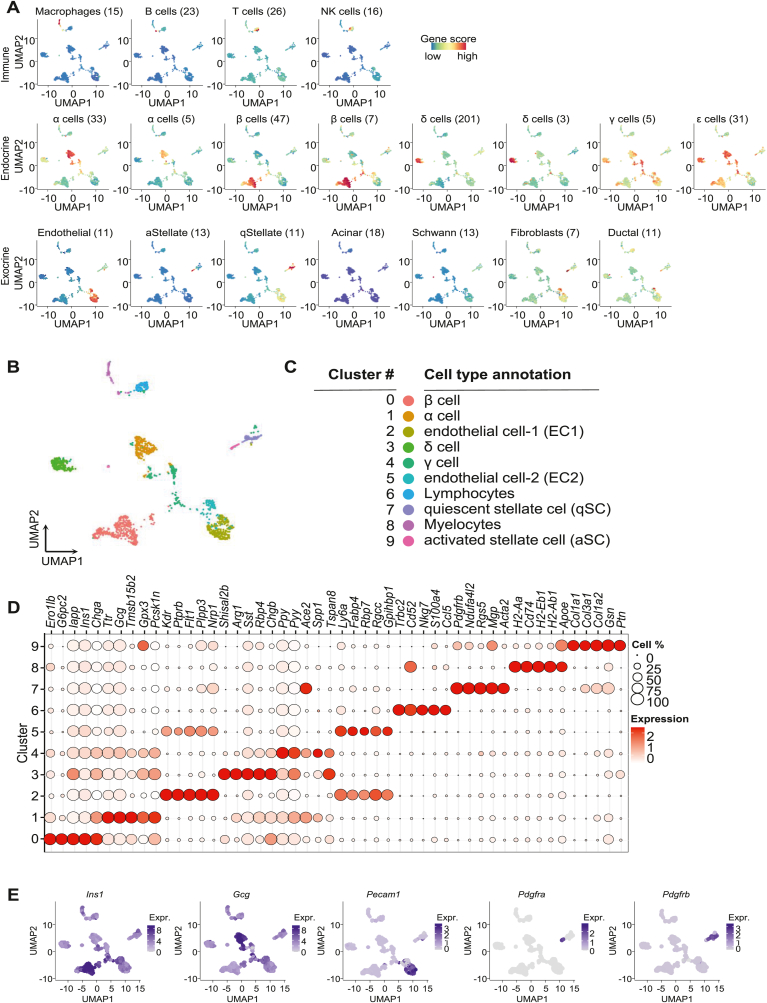
**Identification of distinct endocrine and exocrine cellular subsets in murine pancreas.** (**A**) Single cell RNA-sequencing (scRNA-seq) data of 1680 pancreas-resident cells visualized by non-linear uniform manifold approximation and projection (UMAP) dimensionality reduction and gene module scoring for immune (upper panel), endocrine (middle panel), and exocrine (lower panel) cell populations. Gene lists used for module scorings are provided in Suppl. Table 1). (**B**) scRNA-seq data of pancreatic cells visualized by UMAP dimensionality reduction and coupled to Louvain clustering, revealing ten distinct cell clusters. (**C**) Cell type-specific annotation of pancreas-resident cell clusters based on module scoring as shown in panel A, for endocrine (α-, β-, γ-, and δ-cells), exocrine (two endothelial clusters [EC1, EC2], qSCs and aSCs), and immune (lymphoid, myeloid) cells. Genes lists used for scoring and respective sources are provided in Supplementary Table 1. (**D**) Top marker genes for each of the 10 Louvain clustering-identified cell populations. Gene expression data visualized as dot plots showing the percentage of cells expressing the module signature in each cell cluster, and the mean expression across each cell cluster. (**E**) Established marker genes of four pancreatic cell subsets used to verify cell type annotations. UMAP feature plots showing scaled gene expression in all cells for β-cell marker gene *Ins1*, α-cell marker gene *Gcg*, endothelial cell marker gene *Pecam1*, aSC marker gene *Pdgfra*, and qSC marker gene *Pdgfrb*.

To annotate individual cell subsets with their respective cell type identities, we applied gene module scoring using a collection of nineteen (19) pancreatic cell-type specific gene expression sets of markers for various immune, endocrine, and exocrine cell types (Fig. 1A; Suppl. Fig. S1; Suppl. Table 1) [19,20]. These results showed a very high degree of cluster-specific expression for the different cell type-dependent gene expression signatures, verifying a balanced representation of expected endocrine, exocrine, and immune cell populations in our dataset. After canonical correlation analysis (CCA), the integrated dataset was Louvain-clustered, revealing ten distinct cell clusters which could subsequently be mapped to the initial scoring to assign cellular identifies to each cluster, which included prominent endocrine cells like α- and β-cells, and cells mainly found in exocrine regions, such as two EC clusters (EC1, EC2), as well as aSCs and qSCs (Fig. 1B and C). Cluster-specific gene expression markers were identified by differential gene expression analysis for marker genes specifically enriched in each cell type cluster (Suppl. Tables 2 and 3). These markers included genes such as insulin (*Ins1*) in cluster 0, thus labeling β-cells, the α-cell marker glucagon (*Gcg*) in cluster 1, the EC marker platelet and endothelial cell adhesion molecule-1 (*Pecam1*) in cluster 2, the aSC marker *Pdgfra* in cluster 9, and the qSC marker *Pdgfrb* in cluster 7 (Fig. 1D and E).

**Fig. 2:**
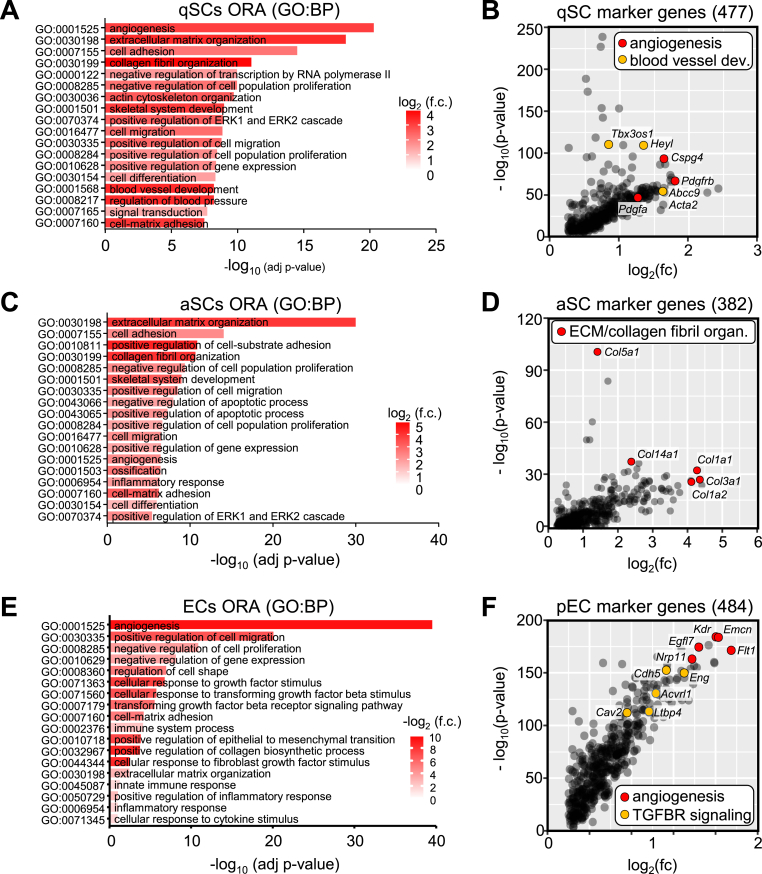
**Pancreatic stellate and endothelial cells jointly express ECM- and angiogenic gene signatures**. (**A**) Top 18 significantly enriched pathway terms from ORA using GO:BP of genes enriched in qSCs. (**B**) Correlation analysis of the 477 qSC-specific marker genes plotting the -log10 p-value (y-axis) against log2 fold-change (x-axis) for individual genes compared to all other cells in the dataset. Colored dots indicate top 5 genes either linked to the GO:BP term angiogenesis (GO:0001525; red) or the term blood vessel development (GO:0001568; yellow), including qSC marker gene *Pdgfrb*. (**C**) Top 18 significantly enriched pathway terms from ORA using GO:BP of genes enriched in aSCs. (**D**) Correlation analysis of the 382 aSC-specific marker genes plotting the -log10 p-value (y-axis) against log2 fold-change (x-axis) for individual genes compared to all other cells in the dataset. Red dots indicate top 5 genes linked to the GO:BP terms extracellular matrix organization (GO:0030198) and collagen fibril organization (GO:0030199). (**E**) Top 18 significantly enriched pathway terms from ORA using GO:BP of genes enriched in ECs, from combined clusters EC1 and EC2. (**D**) Correlation analysis of the 484 EC-specific marker genes plotting the -log10 p-value (y-axis) against log2 fold-change (x-axis) for individual genes compared to all other cells in the dataset. Colored dots indicate top 5 genes either linked to either of the GO:BP term angiogenesis (GO:0001525; red) or transforming growth factor beta receptor (TGFBR) signaling pathway (GO:0007179; yellow).

To more comprehensively assess the cell type-specific functions, we used all marker genes for the three cellular subsets, qSCs (475 markers genes), aSCs (382 marker genes), and a list representing marker genes for combined EC-clusters 2 and 5 (484 marker genes; Suppl. Table 3) for overrepresentation analysis (ORA) of gene ontology terms for biological processes (GO:BP). For qSCs, these analyses showed an enrichment for ontologies related to angiogenesis (terms GO:0001525, GO:0001568, GO:0008217) and ECM processes (terms GO:0030198, GO:0030199, and GO:0007160; Fig. 2A). This was also confirmed on the individual marker gene level, as genes of the terms ‘angiogenesis’ and ‘blood vessel development’ were among the most significantly enriched individual genes for qSCs (Fig. 2B). To explore the potential mechanisms of cellular crosstalk, we next applied a database tool to identify potentially secreted gene products among these marker genes [21]. ORA of only these putatively secreted genes confirmed the enrichment of terms linked to ECM and vascularization, suggesting that qSCs may interact with ECs in a paracrine manner to regulate angiogenesis (Supp. Fig. S2A and S2B). We then conducted similar analyses in aSCs, which showed similar enrichment of ECM-related GO terms, while angiogenesis-related terms were either absent or featured much lower significance scores (Fig. 2C). Among the mostly highly enriched individual genes, we found several collagen-encoding genes (Fig. 2D). These observations further support the conclusion that aSCs are more directly linked to ECM-formation and fibrosis, whereas qSC rather assume an interactive role with ECs to maintain vascularization. Secreted gene prediction analysis of aSCs yielded similar results (Suppl. Fig. S2C). We lastly examined overrepresented terms in all ECs of the combined clusters 2 and 5, which featured angiogenesis-related terms (GO:0001525 and GO:0010718), but also terms linked to growth factor signaling-related terms, including TGFB (GO:0071363, GO:0071560, and GO:0007179; Fig. 2E and F). Corresponding analyses of the predicted secretome confirmed these observations, again highlighted the possibility of a close paracrine interaction between ECs and SCs (Suppl. Fig. S2D). To gain further insights into pro-fibrotic ECM processes stemming from the physical interactome of these cell type-specific gene products we used the markers genes identified from our scRNA-seq dataset for a protein-protein interaction enrichment analysis (PPI). For qSCs, this *in silico* analysis yielded the terms ‘collagen biosynthesis and modifying enzymes’ and ‘vascular process in circulatory system’, emphasizing the interpretation that this subset of SCs is involved in regulation of angiogenesis alongside ECM-production (Suppl. Fig. S2E). Conversely, when analyzing aSCs, only the term related to collagen biosynthesis, but not angiogenesis, was retained (Suppl. Fig. S2F). Of note, while multiple collagens feature in both analyses, the gene encoding for the fibrillar type-I collagen, *Col1a1*, was only detected in aSCs. Since this collagen type is considered the main component of fibrotic ECM-deposition [10], this observation supports the interpretation that aSCs are the main component involved in formation of fibrosis.

### 2.2 Aging promotes a pro-fibrotic transcriptomic profile in activated stellate cells

**Fig. 3:**
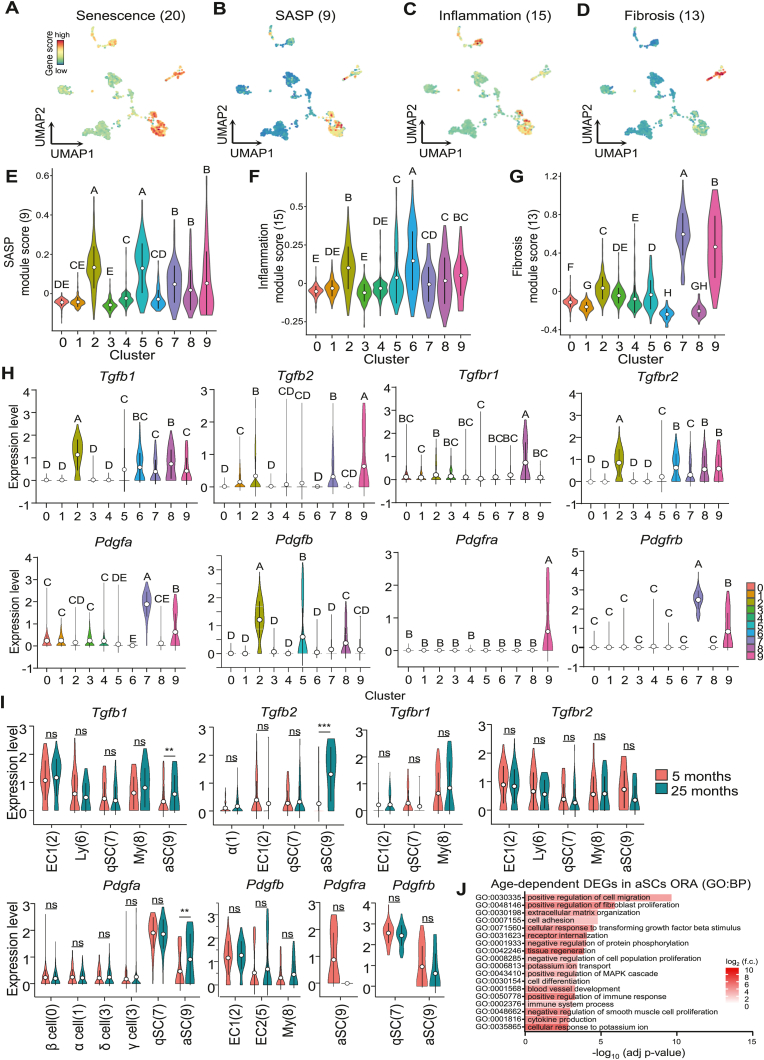
**Pancreatic ECs and SCs display gene signatures related to senescence, inflammation and fibrosis**. (**A-D**) Gene module scoring for marker genes of senescence (A), SASP (B), inflammation (C), and fibrosis (D) visualized as UMAP dimensionality reduction of scRNA-seq dataset (marker genes as specified in Suppl. Table 4). (**E-G**) Density curve visualization of cluster-specific gene module scoring distributions throughout the ten previously identified cell type-specific Louvain clusters for marker genes of SASP (E), inflammation (F), and fibrosis (G) within scRNA-seq dataset (marker genes as specified in Suppl. Table 4). Open circles represent mean values of distribution, error bars represent standard deviation (SD) from mean distribution. Statistical significance was tested by one-way analysis of variance (1way-ANOVA), where absence of similar letters indicates statistically significant differences, and mean expression from high to low is indicated alphabetically. (**H**) Louvain cluster/cell type-specific expression distribution of indicated genes related to TGFB signaling (*Tgfb1*, *Tgfb2*, *Tgfbr1*, *Tgfbr2*) and PDFG signaling (*Pdgfa*, *Pdgfb*, *Pdgfra*, *Pdgfrb*). Open circles represent mean values of distribution, error bars represent standard deviation (SD) from mean distribution. Statistical significance was tested by one-way analysis of variance (1way-ANOVA), where absence of similar letters indicates statistically significant differences, and mean expression from high to low is indicated alphabetically. (**I**) Age-dependent expression distribution analysis of the indicated genes in the indicated clusters (EC1; aSCs; qSCs; Ly – lymphoid cells; My – myeloid cells; α – alpha cells) comparing cells derived from pancreata of adult, 5-months old mice (red) to cells derived from very aged, 25-months old mice (green). Open circles represent mean values of gene expression, error bars represent standard deviation (SD) from mean distribution. Statistical significance was assessed by T-test with Welch's correction, significant differences are indicated as ∗∗*P* < 0.01, ∗∗∗*P* < 0.001. (**J**) Top 18 significantly enriched pathway terms from ORA of DEGs using GO:BP and comparing adult aSCs to aged aSCs.

To examine age-relate changes in our scRNA-seq dataset, we repeated module scoring with gene lists representing senescence, the senescence-associated secretory phenotype (SASP), inflammation and fibrosis (Supp. Table 3). These analyses showed that SCs and ECs were the main cell clusters enriched with senescence-related transcripts and inflammatory processes (Fig. 3A–H; Suppl. Fig. S3A–S3B). Several immune cell populations scored highly for inflammation alongside ECs and SCs, while SCs displayed the highest module score for fibrosis (Fig. 3G and H). Although we observed an increase in SASP scoring for the combined endothelial cell clusters 2 and 5 with age (Suppl. Fig. S3C), somewhat unexpectedly, no difference was found between cells of adult (5-months) and very aged (25-months) mice for all scores per cluster, suggesting that these aging-related marker gene sets may not be universally applicable and that the cell types in question exhibit high baseline gene expression signatures reflecting phenotypes typical for senescence, such as cell cycle arrest and expression of inflammatory regulators (Suppl. Fig. S3D–K). In line with these findings, EC clusters 2 and 5, as well as SC clusters 7 and 9 displayed the lowest scores for actively cycling cells, i.e. S and G2M cell cycle phases, also showing no difference between cells collected from adult and very aged mice (Suppl. Figs. S3I, S3J, S3K, S3L). Therefore, and related to the observation of growth factor-mediated crosstalk between ECs and SCs, we also scored for the individual genes encoding TGF- and PDGF-signaling ligands and receptors. Genes encoding for TGF ligands, *Tgfb1* and *Tgfb2*, as well as both receptor genes, *Tgfbr1* and *Tgfbr2*, were readily detected in ECs, aSCs, and qSCs (Fig. 3H). For PDGF-signaling components, *Pdgfa* was mainly observed in both SC populations but was much lower expressed in most other pancreatic cell clusters. Conversely, *Pdgfb* was mainly expressed in ECs, and at a significantly lower level in the other clusters. PDGF receptor genes displayed the highest cell type-specificity, with *Pdgfra* being readily detected in the aSC cluster and *Pdgfrb* showing highest expression in the qSCs cluster. These data are consistent with previous reports that *Pdgfra* is mainly expressed in activated over quiescent SCs in pancreatic tissue [19]. For those clusters which displayed detectable levels of these eight genes, we next examined age-related differences and found that only aSCs showed significant inductions by age for three out of four ligand-encoding genes, *Tgfb1*, *Tgbf2* and *Pdgfa* (Fig. 3I). None of the other cell subsets with high expression levels showed significant differences in gene expression between the two age groups, altogether suggesting that aging mainly affects aSCs in our scRNA-seq dataset. When assessing over-represented pathways in the genes with significantly higher expression in aged compared to young adult aSCs, terms related to the ECM were upregulated, e.g. ‘positive regulation of fibroblast proliferation’ (GO:0048146) and ‘ECM organization’ (GO0030198) alongside the term ‘cellular response to transforming growth factor beta stimulus’ (GO:0071560), indicating that aged aSCs feature a pro-fibrogenic transcriptional signature (Fig. 3J). Further supporting the pro-fibrotic role of aSCs in the aging pancreatic niche, analysis of disease-related Medical Subject Headings (MeSH) terms associated of differentially expressed genes (DEGs) upon aging in aSCs found several significantly enriched terms including inflammation, fibrosis, atherosclerosis, and carcinogenesis, all of which are linked to a pro-fibrotic environment (Suppl. Fig. S4A). To better evaluate age-dependent pro-fibrotic changes in the cellular crosstalk between ECs and SCs, we conducted an *in-silico* analysis of intercellular communications [22]. When analyzing the entirety of upregulated ligand-receptor interactions (LRIs) with age in our dataset, *Tgfb1*-linked signaling was among the top significant LRIs, and was specifically directed from aSCs and ECs towards quiescent stellate cells (Suppl. Fig. S4B, Suppl. Table 6). In contrast, *Tgfb2*-linked signaling was directed primarily from aSCs towards endocrine α-, β-, and γ-cells, suggesting an age-dependent upregulated bifurcation of TGFB signaling based on specific ligand-receptor combinations, with the profibrotic SC-activating TGFB1 ligand primarily directed towards qSCs (Suppl. Fig. S4C). Moreover, characterization of upregulated LRI-depended KEGG pathways with age across all cells in our dataset identified fibrosis-associated KEGG pathways including cellular senescence, pancreatic cancer, and TGFB signaling, further supporting a shift of LRI-depended profibrotic *Tgfb1*-and *Tgfb2*-linked alterations (Suppl. Fig. S4D). Gene-Term association analysis [23] of TGFB- and PDGF-signalling genes to pancreatic fibrosis and cancer confirmed the primary role of the TGFB1-TGFBR2 genes in the field of pancreatic fibrosis and pancreatic cancer (Suppl. Fig. S4E). In summary, this analysis supports the conclusion that SC-related cellular crosstalk and SCs themselves are main source of ECM and pro-fibrogenic signals in the pancreas.

### 2.3 Aged murine pancreas displays increased adipocyte infiltration and fibrosis in distinct microanatomical locations

**Fig. 4:**
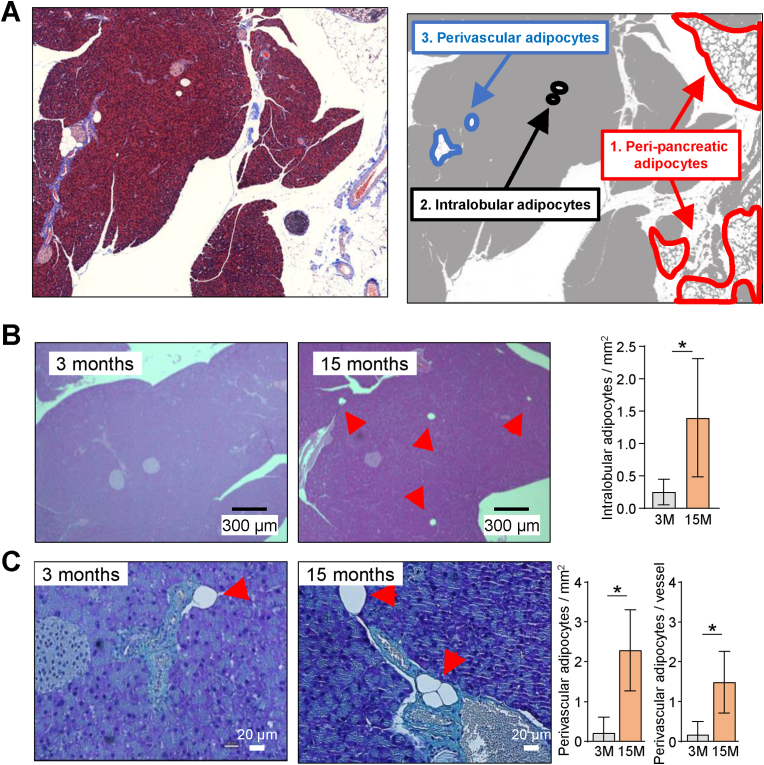
**Age-dependent accumulation of adipocytes occurs in distinct areas of the exocrine pancreas.** (**A**) Representative Masson's trichrome staining of pancreas histological section from an adult male C57Bl/6J mouse aged 5 months. Blue stained areas indicate collagen-rich deposits, purple/red staining indicates cytosolic regions of lobular exocrine pancreas, light pink indicated islets, dark purple/black dots indicate nuclei, and white, non-stained circular areas indicate adipocytes (left panel). Diagram in red panel outlines three main types of adipocytes by microanatomical localization, indicating larger peri-pancreatic adipocyte depots (red outlines), individual intralobular adipocytes (black outlines), and smaller depots of perivascular adipocytes (blue outlines). (**B, C**) Representative hematoxylin and eosin (H&E) stainings and quantifications of adipocytes in pancreas histological sections from young adult (3 months) and aged (15 months) mice (red arrows indicate adipocytes). Quantifications show intralobular adipocytes (B), or perivascular adipocytes (C), calculated as percentages (%) of total tissue area. Data are depicted as means, error bars indicate standard errors of the mean (SEM; n = 4). Statistical significance was assessed by T-test with Welch's correction, significant differences are indicated as ∗ *P* < 0.05.

In previous analyses of pancreatic SCs we had observed a robust adipogenic in vitro differentiation potential, suggesting that SCs may give rise to pancreatic adipocytes [24]. Previous studies have reported on increased steatosis, also known as fatty infiltration, and fibrosis, as morphological hallmarks of the aging pancreas [7,25]. To verify these findings in our animal model, we conducted a histological assessment of adipocyte accumulation and fibrosis deposition in pancreata collected from young adult and aged mice. Bona fide adipocytes were found in distinct locations of the pancreas, locating either to large peri-pancreatic depots broadly surrounding the organ itself, or to intra-pancreatic regions. These latter adipocytes were interspersed throughout the exocrine tissue but not within islets, either as individual cells or small groups of very few individual cells, which are termed intralobular adipocytes [8]. Additionally, individual cells or small groups were observed in direct proximity to blood vessels, i.e. were termed perivascular adipocytes (Fig. 4A, Suppl. Fig. S5A). In both regions, adipocytes were found to be significantly increased in aged, 15-months old mice compared to young adult mice (Fig. 4B and C).

**Fig. 5:**
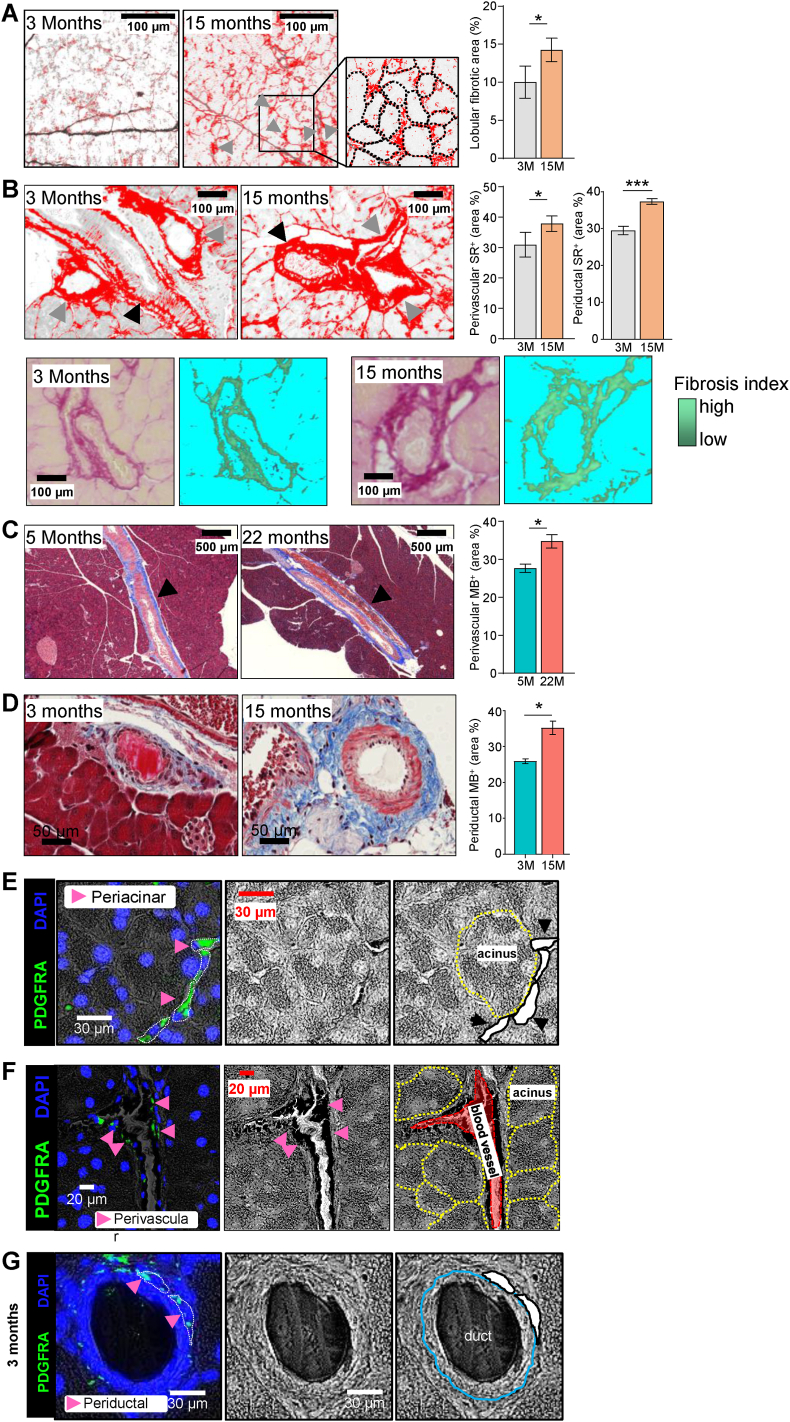
**Aging promotes exocrine pancreas fibrosis that co-localizes with pancreatic stellate cells.** (**A**) Representative Sirius red staining of histological sections of lobular pancreas regions and quantification of lobular Sirius red-positive fibrotic deposits (red staining, black arrows) comparing young adult (3 months) and aged (15 months) mice. Middle panel: representative 100 μm^2^ area with individual acini outlined manually (dotted black line) based on visible boundaries in light microscopic images. (**B**) Representative Sirius red staining of histological sections of perivascular and periductal pancreas regions and quantifications of Sirius red-positive fibrotic deposits (black arrows: perivascular; grey arrows: periductal) comparing young (3 months) and aged (15 months) mice. Lower panels: Periductal regions shown as representative Sirius Red stains (light microscopy) and calculated fibrosis index (right) comparing young adult (3 months) and aged (15 months) mice. (**C**) Representative Methyl blue staining of histological sections of perivascular pancreas regions and quantification of perivascular Methyl blue-positive staining of fibrotic deposits (black arrows) comparing adult (5 months) and very aged (22 months) mice. (**D**) Representative Methyl blue staining of histological sections of periductal pancreas regions and quantification of periductal Methyl blue-positive staining of fibrotic deposits comparing young adult (5 months) and very aged (22 months) mice. All quantifications are calculated as percentages (%) of total tissue area. Data are depicted as means, error bars indicate standard errors of the mean (SEM; n = 4). Statistical significance was assessed by T-test with Welch's correction, significant differences are indicated as ∗ *P* < 0.05. (**E-G**) Immunofluorescence analysis of PDGFRα expression in periacinar (E), perivascular (F), and periductal (G) areas of histological pancreas sections. Green indicates PDGFRα^+^ expression, blue indicates nuclei by DAPI staining, pink arrows indicate SCs, based on morphological assessment of bight field microscopic images (middle panels), with structures, i.e. individual structures colour-marked (acini – yellow; blood vessel – red, duct – blue, SC – white areas with black borders) in corresponding panels on right.

Microscopic assessment of fibrotic depositions in the lobular exocrine regions of the pancreas showed a layer of collagen-rich ECM surrounding individual acini (Suppl. Fig. S5A). More pronounced ECM-layers in these areas were observed in aged pancreas, which resulted in significant increases of fibrotic depositions in aged pancreas tissue upon quantification (Fig. 5A). We also noticed particularly pronounced areas of collagen-rich, fibrous matrix surrounding blood vessels and pancreatic ducts, and quantified these areas independently of the lobular and peri-acinar fibrosis areas. As before, aging resulted in a significant increase of these perivascular and periductal fibrotic depositions in two different cohorts of aging mice (Fig. 5B–D). Since SCs have been proposed as the main source of both adipocytes and ECM within pancreas, we next determined whether SCs were indeed present in these distinct regions as our initial scRNA-seq was performed on islet-enriched tissue samples. We found that SCs marked by expression of PDGFRα were readily detectable in pancreata of mice at all ages used in this analysis directly adjacent to acini as well as in perivascular and periductal areas (Fig. 5E–G; Suppl. Fig. S5B and S5C). Notably, the presence of SCs has been reported both around and within islets [26]. These observations taken together establish that SCs indeed occur in the regions of the pancreas that typically feature high levels of adipocyte formation and fibrous ECM depositions.

### 2.4 scRNA-seq of activated pancreatic stellate cells shows that aging results in their pro-fibrogenic commitment and differentiation

**Fig. 6:**
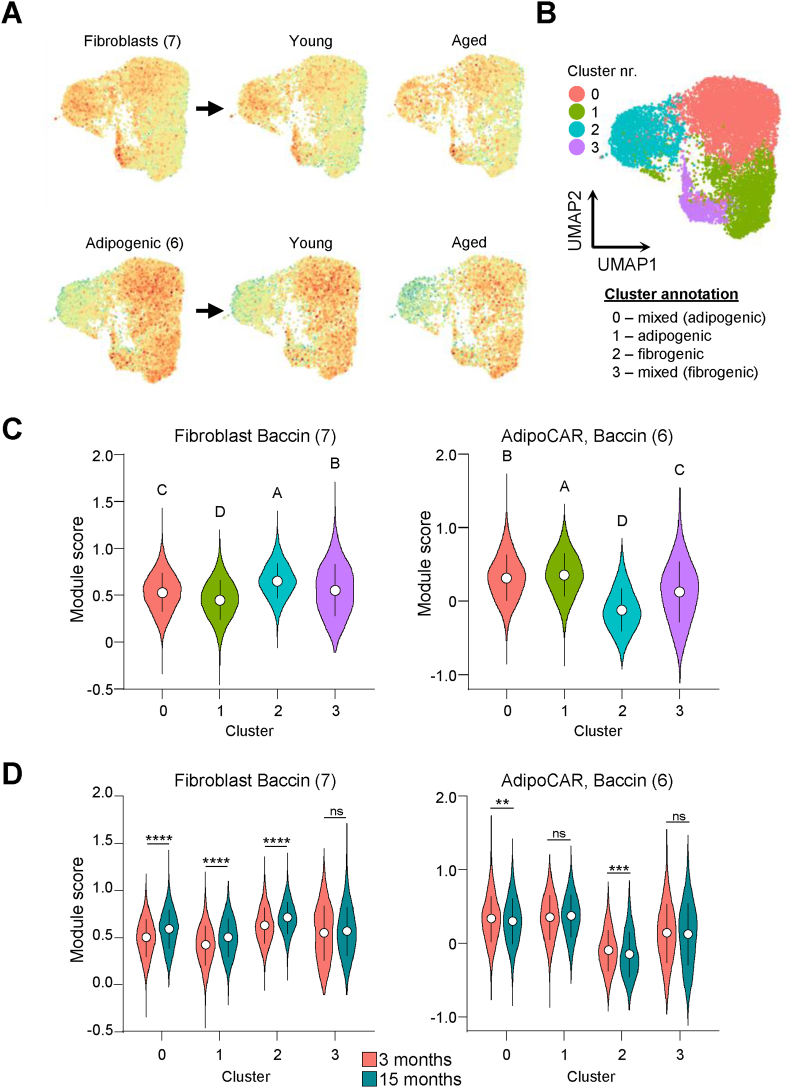
**Pro-fibrotic specialization of PDGFRα^+^ SCs at the transcriptional level increases during aging.** (**A**) UMAP dimensionality reduction projection coupled to module scoring for fibroblast and adipogenic gene expression signatures of scRNA-seq data of 11,839 pancreas-derived, FACS-purified PDGFRα^+^ aSCs isolated from young adult (3 months) and aged (15 months) mice. (**B, C**) UMAP visualization (B) and module score distribution for fibroblasts (C, left) or adipogenic genes (C, right) of unsupervised Louvain clustering into 4 distinct clusters, and subsequent cluster annotation based on module scoring results into transcriptionally distinct clusters with mixed expression profile clusters 0 and 3, which show propensity towards either adipogenic or fibrogenic expression signatures, respectively, and the decidedly adipogenic cluster 1 and the decidedly fibrogenic cluster 2. Open circles in panel C represent mean values of module score distribution, error bars represent SD. Statistical significance was tested by one-way analysis of variance (1way-ANOVA), where absence of similar letters indicates statistically significant differences, and mean expression from high to low is indicated alphabetically. (**D**) Age-dependent expression distribution analysis of the indicated module scores (fibroblast or adipogenic) in the indicated clusters comparing PDGFRα^+^ cells derived from pancreata of young adult, 3-months old mice (red) to cells derived from aged, 15-months old mice (green). Open circles represent mean values of gene expression distribution, error bars represent SD. Statistical significance was assessed by T-test with Welch's correction, significant differences are indicated as ∗∗*P* < 0.01, ∗∗∗*P* < 0.001, ∗∗∗∗*P* < 0.0001, ns – not significant.

To more directly assess age-related transcriptomic differences in aSCs in greater depth, we next conducted a separate scRNA-seq experiment using flow cytometry-purified aSCs by positive selection of PDGFRα^+^ cells. Following filtering of low-quality cells, transcriptomes of 11,839 pancreatic cells were initially generated, with 7288 cells from young adult, 3-months old mice, and 4551 cells from aged, 15-months old mice. Module scoring and unsupervised Louvain clustering initially revealed 7 distinct clusters, but showed that minor contaminations of epithelial-type cells as well as qSCs were present in the dataset, which were subsequently removed to focus on the purified aSC-subset (Suppl. Fig. S6A–D). This depleted dataset of PDGFRα^+^ aSCs showed a high level of homogeneity and a high degree of overlap following dimensionality reduction between the two age groups (Suppl. Fig. S6E). Module scoring, using previously published fibrogenic and adipogenic marker gene sets to characterize functionally distinct mesenchymal stromal cell (MSC) subsets in bone [27], combined with unsupervised Louvain clustering identified 4 distinct clusters. Further inspection of the clusters showed that clusters 0 and 3 were mixed clusters, whereas cluster 1 was enriched for cells expressing an adipogenic marker gene signature and cluster 2 was enriched for cells with a fibrogenic gene expression signature (Fig. 6A and B). Since it is challenging to identify individual differentially expressed genes in a dataset with a large number of individual cell transcriptomes, we also tested module scoring in cells derived from young adult compared to aged mice and found that aging resulted in a significant increase in fibrogenic gene expression in fibrogenic cluster 2, and also clusters 1 (adipogenic) and 0 (mixed), which is consistent with the observation that aging results in increased fibrous deposits in the pancreas. Unexpectedly, we observed no age-dependent differences in adipogenic gene signature in the adipogenic cluster 1, but the signature was significantly reduced in clusters 0 (mixed) and 2 (fibrogenic) (Fig. 6C). Altogether, these observations support the notion of increased pro-fibrogenic lineage commitment of aSCs in the context of aging, whereas the increased adipocyte accumulation observed in the aging pancreas seems unrelated to altered lineage specification in SCs.

### 2.5 Pancreatic stellate cells from aged mice display an increased fibrogenic capacity in vitro

**Fig. 7:**
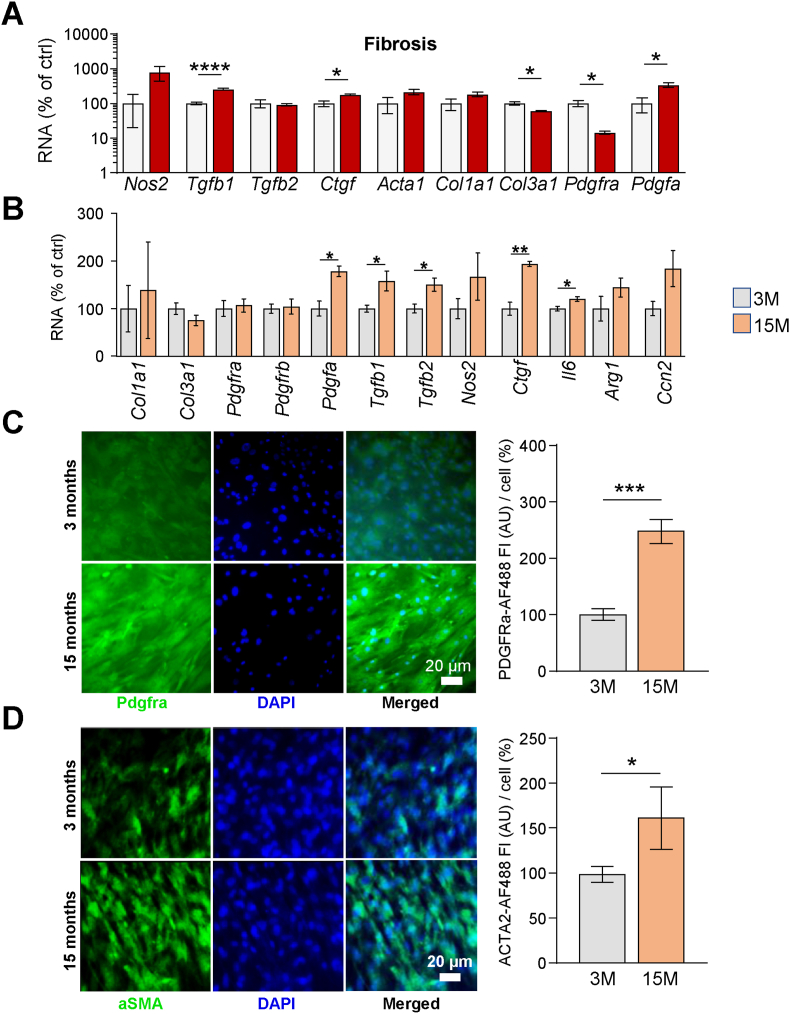
**Aged pancreatic PDGFRα^+^ SCs show increased TGFB1-mediated fibrogenic differentiation potential**. (**A**) mRNA expression analysis of fibrosis marker genes comparing PDGFRα^+^ SCs under baseline culture conditions (grey bars) to TGFB1-exposed culture conditions (red bars) by qPCR. Bars represent the mean ± SEM (n = 3–8), statistical significance was assessed by T-test with Welch's correction, significant differences are indicated as ∗ *P* < 0.05, ∗∗∗∗*P* < 0.0001. (**B**) mRNA expression analysis of fibrosis marker genes comparing in vitro TGFB1-exposed PDGFRα^+^ SCs isolated by flow cytometry from young adult (3 months; grey bars) and aged (15 months; orange bars) mice. Bars represent the mean ± SEM (n = 4–6), statistical significance was assessed by T-test with Welch's correction, significant differences are indicated as ∗ *P* < 0.05, ∗∗*P* < 0.01. (**C**) Representative immunofluorescence images and quantifications of PDGFRα expression in cultured SCs, comparing cells isolated from young adult (3 months; grey bars) and aged (15 months; orange bars) mice, after exposure to TGFB1. Green indicates PDGFRα^+^ cells, blue indicates DAPI^+^ nuclei, merged images are depicted on right. Bars represent the mean ± SEM (n = 7, showing 1 of 3 independent experiments), statistical significance was assessed by T-test with Welch's correction, significant difference is indicated as ∗∗∗*P* < 0.001. (D) Representative immunofluorescence images and quantifications of alpha-smooth muscle actin (aSMA) expression in cultured SCs, comparing cells isolated from young (3 months; grey bars) and aged (15 months; orange bars) mice, after exposure to TGFB1. Green indicates aSMA^+^ cells, blue indicates DAPI^+^ nuclei, merged images are depicted on right. Bars represent the mean ± SEM (n = 5–7, 3 independent experiments), statistical significance was assessed by T-test with Welch's correction, significant difference is indicated as ∗ *P* < 0.05.

To verify the scRNA-seq results on a functional level, we used a similar approach as before to cultivate flow cytometry-purified pancreatic PDGFRα^+^ aSCs in the presence of TGFB1 to stimulate pro-fibrogenic differentiation [24]. While we previously used surface marker stem cell antgen-1 (SCA1), our flow cytometric analysis showed a high degree of overlap between the SCA1+ and the PDGFRα^+^ expressing cells, indicating that both markers could be used to effectively isolate aSCs from the pancreas (Suppl. Fig. S7A–C). Under these conditions, aSCs displayed significant differences in gene expression of fibrosis genes, where typical pro-fibrotic genes such as *Tgfb1*, *Ctgf* (encoding for: connective tissue growth factor) and *Pdgfa* were significantly induced, establishing the pro-fibrotic differentiation potential of aSCs (Fig. 7A). Consistent with the scRNA-seq results, aSCs isolated from aged mice displayed significantly increased expression of several fibrosis marker genes, such as *Pdgfa*, *Tgfb1*, *Tgfb2*, *Ctfg* and *Il6* when compared to aSCs isolated from young adult mice (Fig. 7B). Aged cells also displayed increased expression of fibrosis protein markers PDGFRα [9] and alpha smooth muscle actin (ACTA2) [9] following TGFB1 exposure (Fig. 7C and D).

## 3 Discussion

In our study, we characterize the paracrine crosstalk between the SC- and EC-compartments in the context of pancreas aging. We find that both cells populations feature gene expression patterns reminiscent of quiescence, cell cycle arrest, and senescence, regardless of age. With increasing age, pro-fibrotic pathways, TGFB- and PDGF-signaling, become more pronounced, resulting in altered ECM expression profiles alongside excessive fibrosis deposition in the exocrine regions of the pancreas and adipocyte infiltration. SCs readily display both adipogenic and fibrogenic differentiation under in vitro conditions. Yet, on the cellular level, aging is mainly accompanied by a pro-fibrogenic switch in the transcriptional state of SCs whereas the ability to produce adipocytes appears unaltered by age. These observations suggest the presence of cell-autonomous mechanisms driving pancreatic fibrosis based on a paracrine SC-EC crosstalk, whereas excessive pancreatic fatty infiltration with adipocyte accumulation appears to rely on systemic signals.

SCs of the pancreas are fibroblastic cells phenotypically homologous to MSC-type cells that have been described as resident in many tissue types [28,29]. This cell type is generally considered a main source of ECM proteins, thereby contributing important aspects of tissue maintenance, but also pathological features. Our data suggest that both SC subsets, quiescent and activated, could readily contribute discrete regulatory signals to physiological processes, where qSCs contribute to tissue homeostatic processes, including regulation of angiogenesis and healthy ECM production, whereas prolonged activation into aSCs may contribute to pathological, pro-fibrogenic processes, as observed in the aging pancreas. This is consistent with previous literature contending that activation events of SCs requires inflammation signal input, such as activation of p38-Mitogen activated protein kinase (p38MAPK)-signaling and other related pathways via inflammation-related cytokines [9,30]. Activation in turn results in enhanced ECM production, including type-I collagen, which we also found specifically enriched in the aSC subset [30]. The source of such inflammation-related signals remains unclear and our analyses suggest that a close crosstalk between SCs and the endothelial compartment of the pancreas may contribute to this effect. Pancreatic SCs, feature high and age-independent signatures of senescence, fibrogenesis and inflammation. However, SASP gene expression increases with age collectively in ECs, suggesting ECs promote senescence within the local milieu. Indeed, analysis of the entirety of upregulated LRI-depended KEGG pathways with age also identifies cellular senescence, further supporting the hypothesis that senescence of the pancreatic microenvironment might also materialize at the non-cell autonomous interactome or cell-to-cell communication level, rather than exclusively at the cell-autonomous transcriptomic level. We observe that an age-related onset of TGFB- and PDGF-signaling pathways may stimulate the fibrosis-supporting crosstalk and exacerbate these signatures in aged murine pancreata. These observations taken together suggest that both cell types interact and jointly contribute to some aspects of pancreas tissue aging, ultimately resulting in accumulation of fibrosis and act as a source of fatty infiltration of the tissue. Endothelial senescence has been reported across different organs, supporting the view that it contributes to a variety of pathological processes linked to vascular dysfunction [31,32]. Systemic metabolic pathologies, including hyperglycemia and dyslipidemia, can drive vascular dysfunction but emerging evidence shows that endothelial cell senescence and dysfunction precedes, and often plays a causal role in such conditions [31].

Unexpectedly, we observed increased expression patterns of senescence in adult and aged EC- and SC-subsets. Cellular quiescence is a static state characterized by a lack of proliferation [33], which associates to the cell cycle phases G1 and G0 [34,35]. Our single cell transcriptomics results show that compared to other cell types in the pancreas, ECs and SCs exhibit a low expression of cell cycle and proliferation-associated G2M/S phase transcripts, and a high expression of G1 phase-related transcripts. These findings might suggest some causative link between cell cycling, regeneration, and senescence, but more experimental work is required to confirm this hypothesis. Moreover, removal of pECs from the cell cycle involves common regulatory processes shared by quiescence and senescence [32,36]. This observation also suggests that the contribution of pancreatic ECs to the pancreas milieu is increasingly deregulatory or deleterious with age, in agreement with what is reported in other tissues [31,32]. Canonical SASP factors represent a group of inflammatory cytokines, growth factors, chemokines and reactive oxygen species (ROS) secreted by senescent cells [36]. The role of EC senescence in ROS generation is pivotal, and even in the presence of high oxygen levels in the pancreatic arteriolar vasculature, and unlike most other cell types, ECs rely primarily on anaerobic respiration to prevent ROS by-products [37]. Thus our finding of increased pancreatic EC senescence transcripts in aged mice could be linked to a promotion of ROS generation by ECs, as observed in senescent ECs from other organs [31,32], which in turn can further accelerate cellular senescence, although such a link would have to be experimentally verified. Recently, endothelial cells have been recognized to play a key role in inflammation, being both responsive to cytokine signaling and expressing inflammatory genes [16] supporting the accepted view that ECs can play a significant role in immunocyte chemoattraction, and in modulating the inflammatory state of the pancreatic niche [15,32]. Additionally, other recent studies have demonstrated that the overall phenotype of senescent endothelial cells comprises a pro-inflammatory component across tissues [31]. Consistent with these findings, our analysis found that ECs expressed high mean levels of SASP- and inflammation-linked genes when compared to every other cell type detected in our scRNA-seq data, including SCs, and did so increasingly with age. Conversely, SCs express significantly higher signatures of fibrosis markers which is further enhanced with age, suggesting that this cell type is the main contributor of fibrosis deposits in the aged pancreas. In line with this conclusion, we have previously shown that pancreatic MSC-type cells have a pro-fibrogenic capacity that is distinct to that of other mesenchymal stromal cells such as those found in white adipose tissue [24]. As accumulation of fibrous deposits in the pancreatic niche, and specifically in peri-ductal loci, is a feature of pancreatic ductal adenocarcinoma progression, our results suggest that the pro-fibrotic state of aged SCs may also play an important role in development of certain types of pancreatic cancer [17].

Our study has some limitations. In this study, we use the terms “young adult” (3-months-old), “adult” (5-months-old), “aged” (15-months-old), and “very aged” (≥22-months-old) to refer to the different age groups of the mice analyzed. However, translating these terms to the human condition can be less straightforward. For instance, in terms of chronological age, a 15-months-old mouse (∼450 days) could be equated to a “middle-aged” (45-year-old) rather than “aged” human, as the maximum lifespan of C57Bl6/J mice, defined as the mean age of the longest-lived 10th percentile, is approximately 31 months (∼950 days) [38]. Yet, there are considerable physiometabolic and life cycle differences between mice and humans, including the fact that female C57Bl6/J mice are post-reproductive at approximately 7-months-old (∼210 days), which would be the chronological equivalent of a 21-year-old woman, meaning that chronologically their reproductive lifespan would represent around half of that seen in human females. Therefore, although in this study we adopt the above terminology for different age cohorts, we acknowledge that their precise definition in terms of biological and chronological aging can be subject to debate, which is beyond the scope of this manuscript. Secondly, our study is also limited in terms of the number of time points analyzed, and the utilized age groups cannot be used to fully elucidate how the pro-fibrotic state change of SCs occurs, i.e. at what precise ages, and whether it is a progressive or abrupt change. Moreover, although our cell-cycle analyses across the tested age groups provides no supportive evidence for proliferative senescence at the transcriptional level, the presence or absence of replicative senescence would have to be further investigated using *in vivo* characterization tools, as would that of stress-induced senescence. For instance, replicative senescence or fibrogenic capacity of cells could also be assessed through transplantation of PDGFRA-eGFP-labelled aSCs isolated from young adult or aged mice, followed by assessment of cell numbers and fibrous deposits. Such approaches could further elucidate the cellular contributions of aged pro-fibrotic aSCs to the deteriorating exocrine pancreatic niche. Lastly, although scRNA-seq is a powerful method for the analysis of different tissue-resident cell populations, current sequencing technologies typically quantify poly-adenylated RNA molecules indiscriminately, and cannot discern translation-competent RNAs from those in a translation-incompetent state. Hence, these methods are insensitive to a number of steps cells utilize to modulate gene expression, including microRNA or lncRNA sequestration and ribosomal dynamics. Moreover, scRNA-seq has some known methodological limitations, including low gene detection rate, sample processing and batch effects, and methodology-dependent biases including those stemming from computational analysis approaches, among others [39,40].

In summary, our study points to a key contribution of the crosstalk between pancreatic ECs and SCs, where pro-inflammatory signals derived from ECs may act via direct action on SC in the local niche. Aging escalated the pro-inflammatory quality of this crosstalk which in turn skews commitment of SC towards a pro-fibrogenic state, which in turn enhances fibrous deposits in the tissue.

## 4 Methods

### 4.1 Mouse models

Unless otherwise indicated, male mice of the C57Bl6/J mouse strain (founder animals for colony obtained from Charles River Laboratories, Sulzfeld, Germany) were used in this study. Moreover, the transgenic reporter mouse strain B6.129S4-Pdgfratm11 (EGFP)Sor/J (strain code: 007669; obtained from The Jackson Laboratory, Bar Habor ME, USA), which expresses green fluorescent protein (GFP) under the control of the *Pdgfra* gene promoter was used in this study. All procedures involving animals were in accordance with Animal Welfare legislation and, where applicable, approved by the ethics committee for animal welfare of the State Office of Environment, Health, and Consumer Protection (Federal State of Brandenburg, Germany). Animals were housed in a controlled environment (20 ± 2 °C, 12 h/12 h light/dark cycle) and maintained on a standard diet (Ssniff, Soest, Germany). All studies were conducted with adult mice, at ages ranging from 3 to 25 months, as stated in the corresponding main text descriptions of the results and figure legends.

### 4.2 Flow cytometry and fluorescence-activated cell sorting (FACS)

Flow cytometry and cell sorting were performed on a FACS Aria III cell sorter (BD Biosciences) and analyzed using FlowJo™ Software (BD, Ashland, OR, USA). For heterogeneous islets, 0.5 mg/ml of Collagenase V (Worthington, Lakewood, NJ, USA) was added directly into the pancreas, followed by digestion for 30 min at 37 C° with shaking. After manual islet selection, islets were disaggregated as previously described [41]. For analysis of PDGFRα^+^ cells, whole pancreata were isolated, cut to small pieces and digested using 1 mg/ml Collagenase-II (Worthington) for 30 min at 37 C° with shaking. Samples were passed through a 40 μm cell strainer and centrifugated at 100×*g* for 7 min at 4 °C. The pellet was re-suspended in Ammonium Chloride Potassium (ACK) lysis buffer to eliminate red blood cells and centrifuged again. Cells were re-suspended in sorting buffer consisting of 100 μl Hank's balanced salt solution (HBSS) containing 2 % FBS (Merck, Rahway, NJ, USA; Biochrom, Middlesex, MA, USA; sorting buffer) and stained using anti-mouse antibodies directed against surface proteins cluster of differentiation (CD)-45, CD31, stem cell antigen (SCA)-1, and PDGFRα (BioLegend, San Diego, CA, USA) for 20 min, washed and re-suspended in 100 μl of 10 mg/ml fluorophore-conjugated antibodies in sorting buffer for 30 min at 4 °C. The applied FACS antibodies can be found in the materials table (Suppl. Table 5). Living cells were gated for the accumulation of calcein blue (1:1000 dilution; stock of 1 mg in 215 mL DMSO) and the exclusion of propidium iodide (PI; 1:1000 dilution; stock of 1 μg/mL in distilled water) fluorescence.

### 4.3 Single cell RNA-sequencing (scRNA-seq) sample preparation

For the analysis of pancreas-resident heterogeneous cells, samples were enriched for endocrine cells by manual collection of pancreatic islets, followed by dispase-digestion and resuspension into a single-cell solution. ScRNA-seq was performed using FACS-purified viable pancreas cells were selected by positive Calcein blue uptake (alive cells) and the exclusion of propidium iodide (PI)-positive cells (dead cells), comparing cells isolated from male mice aged either 5 or 25 months. Approximately 3000 sorted cells were pooled from three mice per sample to obtain heterogeneous viable cells (Suppl. Fig. S1A and S1B). For the scRNA-seq analysis of FACS-purified PDGFRα^+^ cells whole pancreata were used as describe above, comparing cells of 3-months to 15-months old male mice (Suppl. Fig. S6A). Approximately 20,000 cells were pooled from eight mice per sample to obtain the final PDGFRα^+^ cell suspensions.

Sorted cells were subsequently loaded onto a droplet-based single cell processing platform (10x Genomics, Leiden, The Netherlands). Cells were processed according to the manufacturer's instructions. In brief, using the gel beads-in-emulsion (GEM) technique, cells were encapsulated using the Chromium Controller platform (PN1000202, 10x Genomics) as per manufacturers' guidelines. Single cell-cDNA libraries were processed according to the Chromium Next GEM Single Cell 3′ Kit v3.1 (PN1000269, 10x Genomics) library construction workflow, and quality-controlled using Bioanalyzer DNA High sensitivity Kit electropherogram tracing (Agilent Technologies Deutschland GmbH, Waldbronn, Germany). Sequencing reads (paired-end (PE)75) were generated by NextSeq-500 (Illumina, San Diego, CA, USA) NGS sequencing (28 (8)56 mode).

### 4.4 scRNA-seq data analysis

Data pre-processing was performed with CellRanger (versions 5.0.0 - 5.0.1, 10x Genomics), including filtering, barcode counting, and unique molecular identifiers UMI counting, followed by alignment to the reference genome GRCH38 using STAR with default parameters (Suppl. Table 5). The R package Seurat (version 4.3.0) was used for feature selection, data integration, expression matrix scaling, and dimensionality reduction and clustering with default parameters, unless stated otherwise. Cells with <200 genes expressed, and >15 % reads mapped to mitochondrial genes and genes expressed in <5 cells were removed. Data were visualized using UMAP dimensionality reduction. Clustering was performed with a resolution of 0.15-0.2. Differential expression analysis was performed using a Wilcoxon Rank Sum test. Gene Scoring was applied as previously reported [42]. Gene annotation and gene ontology (GO) over-representation analysis were performed using DAVID [43], using p < 0.05 and fold change |FC|≥1.5 as cutoff parameters. *In silico* predicted secretome analysis was applied using VerSeDa [21] using the recommended parameters for the detection of (i) secretory signal peptide, (ii) signal peptide cleavage site, (iii) of glycosylphosphatidylinositol (GPI) membrane anchoring sites, (iv) transmembrane domains (TMD) for negative selection, and (v) subcellular location. Protein-protein-interaction (PPI) enrichment analysis was performed as previously described and visualized using Cytoscape [44,45]. Intercellular communication through direct cell-cell interactions (CCI) and age-related CCI-changes, including CCI-dependent KEGG pathways, in the scRNA-seq dataset were identified using scAgeCom [22]. Analysis of over-representation of differentially expressed genes in GO terms [46] or disease-associated MeSH terms was performed as previously described [47].

### 4.5 Histology and image analysis of pancreas sections

For paraffin embedding, whole pancreas samples were dissected and fixed overnight in 4 % formaldehyde (Carl Roth GmbH + Co. KG, Karlsruhe, Germany) at 4 °C, subsequently dehydrated, and embedded in paraffin sections before 2 μm slices were prepared. Consecutive sections were collected for hematoxylin and eosin (H&E), Masson's Trichrome, or Sirius Red staining according to standard histological protocols. To determine fibrosis area, collagenous matrix deposition was stained with Sirius red staining and Masson's Trichrome performed in pancreas tissue deparaffinized slides incubated. Fibrosis area [μm^2^] was quantified using Image J software (NIH, Bethesda, MA, USA) [48] with the freely available plugins “MRI fibrosis tool” and “Color Deconvolution” [49].

### 4.6 Immunohistochemistry

For paraffin embedding, whole pancreata were dissected and fixed overnight in 4 % formaldehyde (Carl Roth) at 4 °C, subsequently dehydrated, and embedded in paraffin sections before 2 μm slices were prepared. Sections were stained with primary antibodies (see Suppl. Table 5), blocked for 1 h at RT in blocking solution, and stained with secondary antibodies (see Suppl. Table 5) diluted at 1:100 in blocking solution overnight at 4 °C in a humidified chamber). Nuclei were counterstained with 300 nM DAPI (BioLegend) in PBS for 5 min. After washing, sections were mounted with Fluoromount G (eBioscience, San Diego, CA, USA) and stains were visualized using a BZ900 Fluorescence Microscope (Keyence).

### 4.7 *In vitro* assays

*In vitro* assays were carried out as previously described [24]. Briefly, after sorting, pancreas SCs were plated (50.000 cells per 1 well of a 24 well plate, or 30.000 when lower yield) in growth medium (GM; DMEM 1 g/l glucose of DMEM 4.5 g/l glucose, 10 % FBS, 1 % penicillin/streptomycin) containing 5 ng/ml bFGF (Sigma, Burlington, MA, USA) and 0.1 % gentamicin (Sigma) for 1 week. For fibrogenic differentiation, cells were treated with 1 ng/ml recombinant hTGFB1 (Peprotech Rocky Hill, Middlesex, NJ, USA) for four days.

### 4.8 Quantitative real-time PCR

RNA was extracted from harvested cells using TRIzol reagent (Fisher Scientific, Pittsburg, Allegheny, PA, USA) and purified using commercially available RNA Miniprep Kit (Zymo Research Irvine, Orange, CA, USA). RNA was transcribed into cDNA with a high capacity cDNA reverse transcription kit (Fisher Scientific). Quantitative real-time PCR (qPCR) was performed using the Biorad CFX384 Real-Time System (Biorad Hercules, Contra Costa, CA, USA) with Maxima™ SYBR™ Green/ROX 2x qPCR Master Mix (Fisher Scientific). Primers were designed as intron spanning sequences to specifically amplify cDNA while excluding potential contaminating genomic DNA. mRNA expression was calculated relative to the mRNA expression of the housekeeping gene *Gapdh* in the same samples.

### 4.9 Immunocytochemistry

Cells were washed three times with PBS and permeabilized with 0.1 % Triton X-100 in PBS for 10 min, followed by blocking with 3 % bovine serum albumin (BSA) in PBS for 1 h. After blocking, cells were incubated with the primary antibody (1:100 dilution, Suppl. Table 5) in 3 % BSA in PBS overnight at 4 °C. On the next day, cells were washed three times with PBS to remove unbound antibodies and then incubated with the secondary antibody (1:500 dilution, Suppl. Table 5) in 3 % BSA in PBS for 1 h in the dark. Cells were then washed twice and then treated with DAPI (300 nM, Biolegend, Suppl. Table 5) in PBS for 5 min in the dark to stain the nuclei. Finally, cells were washed twice and imaged in a in a BZ900 Fluorescence Microscope (Keyence, Elmwood Park, Bergen, NJ, USA).

## CRediT authorship contribution statement

**George A. Soultoukis:** Writing – review & editing, Writing – original draft, Visualization, Validation, Supervision, Software, Resources, Project administration, Methodology, Investigation, Funding acquisition, Formal analysis, Data curation, Conceptualization. **Marina Leer:** Writing – review & editing, Writing – original draft, Visualization, Validation, Software, Resources, Project administration, Methodology, Investigation, Funding acquisition, Formal analysis, Conceptualization. **Richard Kehm:** Resources, Project administration, Methodology, Investigation, Funding acquisition, Formal analysis, Data curation, Conceptualization. **Laura Villacorta:** Resources, Methodology, Investigation, Formal analysis. **Vladimir Benes:** Writing – review & editing, Supervision, Software, Resources, Project administration, Investigation, Formal analysis, Data curation. **Tilman Grune:** Writing – review & editing, Supervision, Resources, Project administration, Funding acquisition, Conceptualization. **Annika Höhn:** Supervision, Resources, Project administration, Methodology, Investigation, Funding acquisition, Conceptualization. **Tim J. Schulz:** Writing – review & editing, Writing – original draft, Visualization, Supervision, Resources, Project administration, Methodology, Investigation, Funding acquisition, Data curation, Conceptualization.

## Grants

This study was supported in part by grants (IDs: 01GI0925, 82DZD03D2G and 82DZD03D03) to the German Center for Diabetes Research (DZD e.V.), which is funded by the German Federal Ministry of Research, Technology and Space (BMFTR), and the State of Brandenburg. This work was supported by grants from the German Research Foundation (DFG) as individual grants and within the Collaborative Research Centre 1444 (SFB 1444; project ID: 427826188, to T.J.S.). A.H. acknowledges support from the DFG (project ID: 441099963). G.S. acknowledges support from internal competitive project grant from the German Institute of Human Nutrition Potsdam-Rehbruecke (DIfE; grant ID: FJW034206).

## Declaration of competing interest

The authors declare that they have no known competing financial interests or personal relationships that could have appeared to influence the work reported in this paper.

## Data Availability

Data will be made available on request.

## Appendix A Supplementary data

Multimedia component 6 Multimedia component 6

Multimedia component 7 Supplementary Figure S1 (related to main Fig. 1) Overview of heterogenous pancreatic cell type scRNA-seq analysis and of distinct transcriptomes in heterogeneous cel**l subsets.** (**A**) Overview of experimental design. Diverse cell populations were isolated from adult (5 months) and very aged (25 months) male C57Bl/6J mice for the analyses shown. To also capture endocrine cell heterogeneity, samples were partially enriched for islets, and alive cells were sorted for scRNA-seq analysis. (**B**) Cell numbers in scRNA-seq data. Bar chart showing the total number of cells for the two age groups. (**C**) Representation of cell populations across age groups. scRNA-seq data of 1680 pancreas resident heterogeneous cells visualized by UMAP dimensionality reduction and age group, belonging to adult (5 months) or very aged (25 months) mice. (**D**) Heatmap-visualization of the top 5 marker genes representing cluster-specific DEGs (Wilcoxon Rank sum test, p < 0.01). Supplementary Figure S2 (related to main Fig. 2, Fig. 3) aSCs express and secrete most collagen type I (*Col1a1*). (**A**) Secretome analysis of quiescent stellate cells (qSCs), activated stellate cells (aSCs), and endothelial cells (ECs). VerSeDa *in silico* analysis showing the predicted secreted (black bars) and predicted non-secreted (grey bars) gene products for the respective cell type-specific marker genes representing cluster-specific DEGs (Wilcoxon Rank sum test, p < 0.01). (**B**) *In silico* predicted secretome over-representation GO:BP analysis for qSCs, showing terms in decreasing order of significance (-log10 p-value, x axis) identifying extracellular matrix (ECM) and angiogenic processes. (C) *In silico* predicted secretome over-representation GO:BP analysis for aSCs, showing terms in decreasing order of significance (-log10 p-value, x axis) identifying extracellular matrix (ECM) and collagen processes. (**D**) *In silico* predicted secretome over-representation GO:BP analysis for ECs, showing terms in decreasing order of significance (-log10 p-value, x axis) identifying cell migration and angiogenic processes. (**E**) Protein-protein-interaction (PPI) network identifies qSC transcriptome role in extracellular matrix and angiogenic terms. Circle plot of over-represented GO:BP terms (left panel), with the top terms ranked by p-value (right panel), and the contributing genes shown for the top two terms. (**F**) PPI network identifies aSC transcriptome role in extracellular matrix terms characterized by fibrous deposit-forming collagen type I (*Col1a1*) interactions. Circle plot of over-represented GO:BP terms (left panel), with the top terms ranked by p-value (right panel), and the contributing genes shown for the top term. Supplementary Figure: S3 Endothelial cells (ECs) and activated stellate cells (SCs) express high senescence and fibrosis transcripts, and low proliferation-associated tr**anscripts.** (**A**) SCs and ECs (clusters #7 and #2) express the highest levels of senescence marker genes, followed by ECs (cluster #2). (**B**) Established marker genes for senescence and inflammation are highly expressed in ECs. UMAP feature plots showing scaled gene expression in all cells, for *Trp 53*, *Rb1*, and *Cdkn1a*. (**C**) ECs (clusters #2 and #5) express the highest levels of SASP marker genes in very aged (25 months) compared to adult (5 months) mice. (**D**) Fibrosis markers express at similar levels in very aged (25 months) and adult (5 months) mice. (**E**) Pro-inflammatory markers express higher in activated (PDGFα^+^) stellate cells (cluster #9) from very aged (25 months) compared to adult (5 months) mice. (**F**) SASP markers express at similar levels in individual clusters from very aged (25 months) and adult (5 months) mice. (**G**) Senescence markers express at similar levels in individual clusters from very aged (25 months) and adult (5 months) mice. (**H**) Inflammation markers express at similar levels in individual clusters from very aged (25 months) and adult (5 months) mice. Gene lists used for module scoring are provided in supplementary material. (I) Distinct call cycle phase gene expression patterns displayed by separate cell types. UMAP plots combined to cell cycle phase annotation for G1, S, and G2M phases. (**J**) Endothelial (clusters #2 and #5) and stellate cells (clusters #7 and #9) have a higher proportion of G1 cell cycle phase. Stacked bar chart shows the percentage (%) of cells for each phase separately (G1, G2M, S) in clusters 0 to 9. (**K**) Cell cycle phase G2M markers (upper panel) express the lowest in stellate cells (clusters #7 and #9) and endothelial cells (clusters #2 and #9) but at similar levels per cluster in very aged (25 months) and adult (5 months) mice (lower panel). (**L**) Cell cycle phase S markers express the lowest in stellate cells (clusters #7 and #9) and endothelial cells (clusters #2 and #9) but at similar levels per cluster in very aged (25 months) and adult (5 months) mice (lower panel). For cell type comparisons, gene module scores are shown as violin plots, with mean values, quartile ranges, and error bars representing SD, and statistical significance by with mean value and error bars representing SD, and significance tested by 1-way ANOVA (letter display indicating statistically significant differences, and mean expression from high to low indicated alphabetically, upper panel). For age comparisons, significance is shown by T-test with Welch's correction (p∗ < 0.05, lower panel). Gene lists used for module scoring are provided in supplementary material. Supplementary Figure S4: pro-fibrotic cell-cell interactions are driven by increased TGFB1 signaling to qSCs du**ring aging** (**A**) Over-representation analysis (ORA) of MeSH disease terms using the transcriptome of aSCs confirms contributions towards inflammation and fibrosis. MeSH terms ranked by p-value and colored by number of contributing genes in each term. (**B**) TGFB-signaling intercellular communication through direct cell-cell interactions (CCI) and age-related CCI-changes, identified using scAgeCom [22]. (**C**) CCI-dependent ligand-receptor (LRI) enrichment during aging. (**D**) LRI-dependent KEGG pathway enrichment during aging. (**E**) Gene-Term association analysis of TGFB- and PDGF-signalling genes to pancreatic fibrosis and cancer. Left panel: heatmap showing gene-specific publication associations. Right panel: correlation analysis between fibrosis and cancer, with p-value representing linear regression analysis. Supplementary Figure S5 (related to main Fig. 5) Periacinar and perivascular PDGFRα^+^ pancreatic stellate cells occupy distinct niches in the pancreas of both adult and **aged mice**. (**A**) Histological annotation of distinct pancreatic compartments. Left panel: representative pancreas Sirious Red stains in a adult (5 months) male C57Bl/6J mouse. Right panel: the same image annotated to indicate the various compartments including blood vessels, endocrine islets, lobules of exocrine acini, intralobular and interlobular exocrine ducts, connective tissue septa and capsules, and non-stained ectopic fat (adipocytes). (**B**) Lobular PDGFRα^+^ pancreatic stellate cells (pSCs) exhibit a peri-acinar localization independent of age. Left panels: representative immunofluorescence imaging of peri-acinar PDGFRα^+^ pSCs showing an overlay of DAPI (blue), PDGFRα (green), and bright field images of an acinar region from adult (5 months), aged (15 months), and very aged (22 months) male C57Bl/6J mice. Middle panel: corresponding bright field images with a single acinus and multiple peri-acinar cell regions (black arrows) delineated. Right panel: corresponding raw bright field images. (**C**) Lobular PDGFRα^+^ pancreatic stellate cells (pSCs) with a peri-vascular localization independent of age. Left panel: representative immunofluorescence imaging of peri-vascular PDGFRα^+^ pSCs (pink arrows) showing an overlay of DAPI (blue), PDGFRα (green), and bright field images of an acinar region from an aged (15 months) male C57Bl/6J mouse. Middle panel: corresponding bright field image indicating PDGFRα^+^ regions (pink arrows). Right panel: corresponding bright field image with a single blood vessel (red line) and multiple acinar regions (yellow lines) delineated. Supplementary Figure S6: PDGFRα^+^ pancreatic stellate cell subsets are similarly represented in young and **aged mice.** (**A**) Activated (PDGFRα^+^) stellate cells (aSCs) were isolated from young (3 months) and aged (15 months) male C57Bl/6J mice for the analyses shown. (**B**) Distinct gene expression signatures displayed by separate subsets of aSCs. Single cell RNA-sequencing (scRNA-seq) data of purified activated (PDGFRα^+^) stellate cells visualized by UMAP dimensionality reduction and gene module scoring for quiescent, activated, epithelial, fibroblast, and adipogenic markers. Gene lists used for module scorings are provided as supplementary information. (**C**) scRNA-seq data of FACS-purified activated (PDGFRα^+^) stellate cells visualized by UMAP for young (3 months) and aged (15 months) male C57Bl/6J mice. (**D**) scRNA-seq data of FACS-purified activated (PDGFRα^+^) stellate cells visualized by UMAP dimensionality reduction and coupled to clustering. Unsupervised Louvain clustering analysis identified 6 distinct clusters, which are shown color-coded. (**E**) Bar chart of scRNA-seq analysis showing the number of cells across the two age groups (young/3 months, aged/15 months), normalized to the number of total cell input. (**F**) scRNA-seq data of FACS-purified activated (PDGFRα^+^) stellate cells filtered to remove non-aSCs and visualized by UMAP for young (3 months) and aged (15 months) male C57Bl/6J mice. (**G**) Activated (PDGFRα^+^) stellate cells visualized by UMAP dimensionality reduction and coupled to clustering. Unsupervised Louvain clustering analysis identified 4 distinct clusters, which are shown color-coded. Supplementary Figure S7: PDGFRα^+^ pancreatic stellate cells are predominantly SCA-1^+^. (A) Selection of non-endothelial, non-hematopoietic (CD31^−^;CD45^-^) activated (PDGFRα^+^) stellate cells for in vitro analyses. Cells were FACS-isolated from pancreata of 3-months old male C57Bl/6J mice, using negative selection for surface proteins CD31 and CD45 and positive selection for surface protein PDGFRα. Gate shown is preceded by removal of debris, doublets, and dead cells (gates not shown). **(B)** Flow cytometric analysis of PDGFRα^+^ stellate cells from male C57Bl/6J mice pre-selected using surface marker configurations CD45^−^;CD31^-^;SCA1^+^ (SCA1^+^) and CD45^−^;CD31^-^;SCA1^-^ (SCA1^-^) followed by analysis of PDGFRα expression (FACS density plots on left), with quantifications (panels on right) showing high co-expression of PDGFRα mainly in SCA1^+^ cells. Gates shown are preceded by removal of debris, doublets, and dead cells (gates not shown). Bar chart quantifications show significance by T-test with Welch's correction (p∗∗∗∗ < 0.0001). **(C)** Flow cytometric analysis of SCA1 expression in stellate cells isolated from male transgenic mice which express GFP under control of the *Pdgfra* gene promoter. Cells were pre-selected by flow cytometry using surface marker configurations CD45^−^;CD31^-^;SCA1^+^ and CD45^−^;CD31^-^;SCA1^-^ followed by analysis of GFP expression, thus labeling GFP^+^ as PDGFRα^+^ cells (FACS density plots on left), with quantifications (panels on right) showing high co-expression of GFP mainly in SCA1^+^ cells. Bar chart quantifications show significance by T-test with Welch's correction (p∗∗∗∗ < 0.0001). Multimedia component 7
