# Supplementary material for: Pancreatic stellate cells have adipogenic and fibrogenic potentials but only show increased pro-fibrogenic propensity upon aging
Source: Redox Biol. 2025 Jul 29;86:103791. doi: 10.1016/j.redox.2025.103791 (PMC12337652; doi:10.1016/j.redox.2025.103791)
Supplement: Multimedia component 1 [file mmc1.docx]

| **Cell type** | **Marker gene** | **Reference** |
| --- | --- | --- |
| Macrophages | *Adgre1, Cd68, Cd14, Itgam, Csf1r, Ly6g6e, Ly6c2, Itgal, Anpep, Fcgr3, Fcgr1, Cd80, Cd86, Lamp1, Lamp2.* | Zhang et al. 2019, C. Tabula Muris, et al. 2018 |
| B_cells | *Cd19, Cd79a, Cd79b, Il7r, Cd22, Ptprc, Cd2, Cd5, Ms4a1, Cr2, Fcer2a, Il2ra, Cd40, Cd69, Cd80, Cd86, Cd93, Tnfrsf9, Cd70, Tnfsf4, Tnfrsf13b, Tnfrsf13c, Pdcd1.* | Zhang et al. 2019, C. Tabula Muris, et al. 2018 |
| T_cells | *Cd3d, Cd3e, Cd4, Itga2, Il2ra, Cd69, Cd1d1, Il7r, Cd8a, Cd5l, Cd8b1, Cd27, Cd28, Entpd1, Cd44, Sell, Nt5e, Il6st, Tnfrsf4, Tnfrsf9, Ctla4, Cd40lg, Ccr7, Btla, Icos, Pdcd1.* | Zhang et al. 2019, C. Tabula Muris, et al. 2018 |
| NK_cells | *Ncam1, Itgam, Itgax, Fcgr3, Il2ra, Ptprc, Itga2, Cd69, Klrd1, Il2rb, Cd160, Cd244, Klrb1c, Klrk1, Ncr1, Slamf6.* | Zhang et al. 2019, C. Tabula Muris, et al. 2018 |
| α_cells_33 | *Mctp2, Sgce, Irx1, Peg10, Pdk4, Gcg, Arx, Rcn3, Ndst4, Pyy, Avpr1b, Klhl13, Eef1a2, Edn3, Higd1a, Vwde, Irx2, Slc38a5, Ptprk, Mafb, Ace2, Kcnk3, 1700086L19Rik, Wnk3, Dbpht2, Calb1, Etv1, Oxtr, Smarca1, Nudt11, Gria2, Ptprd.* | Baron et al. 2016 |
| α_cells_5 | *Gcg, Irx2, Irx1, Arx, Mafb.* | Baron et al. 2016 |
| β_cells_47 | *Akr1c14, Atf5, Atp2a2, Bace2, Car10, Cbs, Cntfr, Cox6a2, Emilin1, Entpd3, Ero1lb, Fmo1, G6pc2, Gabbr2, Gad1, Gjd2, Glp1r, Gng12, Hapln4, Iapp, Ins1, Ins2, Lrrc10b, Mafa, Nkx6-1, Ntrk2, Nup93, Papss2, Pclo, Pde5a, Phactr1, Pip5k1b, Ppp1r1a, Prlr, Prss53, Robo1, Slc2a2, Slc4a10, Spc25, Sytl4, Tmem163, Tmem215, Tmem65, Trpm5, Ttc28, Ttyh1, Ucn3.* | Baron et al. 2016 |
| β_cells_7 | *Iapp, Ins1, Ins2, Mafa, Nkx6-1, Slc2a2, Ucn3.* | Baron et al. 2016 |
| γ_cells_5 | *Ppy, Stmn2, Arx, Meis2, Aqp3t.* | Baron et al. 2016 |
| δ_cells_210 | *1110017F19Rik, 1190002N15Rik, 1700091E21Rik, 2610203C20Rik, 4833420G17Rik, 4930529M08Rik, 4930539E08Rik, 9130017N09Rik, AC102815.1, AC151836.1, Accn1, Ache, Ambp, Ankrd34b, Arg1, Arhgap29, Arhgap36, Asph, Avpr1a, AW551984, Bambi, Basp1, BC017612, BC030476, Bcam, Bmp3, Bmp6, Cacna1h, Cacna2d2, Cadm1, Caly, Capsl, Cbln4, Cd164l2, Cd24a, Cd97, Cdc42ep4, Cdhr2, Cdo1, Cela1, Cer1, Cercam, Cidea, Clcn5, Cldn6, Cldn9, Clmp, Clvs1, Cntnap5b, Cpa2, Ctsh, Ctxn1, Cx3cl1, Cys1, Dapk1, Dennd2a, Dgkb, Dlc1, Dlk1, Dnajc22, Dnase2a, Dos, Dpp6, Ednra, Efnb3, Ehf, Erf, F730043M19Rik, Fam107b, Fam149a, Fam81a, Fbxo30, Fermt1, Fgf14, Fgl2, Fstl5, Fxyd6, Gabrb3, Galnt4, Gap43, Gc, Gfra1, Ghsr, Glis1, Gm15725, Gm5406, Gm609, Gnb4, Gng2, Golga7b, Gpm6a, Gpr179, Gsta4, Gstm7, Hectd2, Hgf, Hhex, Hlf, Hpgd, Hsd17b11, Hspb1, Kbtbd11, Kcna5, Kctd8, Klhdc8b, Lats2, Ldlrad3, Lhfpl2, Lmo3, Lrp1, Lrrc8b, Ly6h, Lypd1, Mboat1, Mdga2, Meox1, Mest, Met, Mfge8, Mpp2, Nab2, Ncam2, Ncs1, Neurl1a, Neurog3, Ngb, Nostrin, Nptx1, Nrip1, Nrsn1, Nsg1, Ntm, O3far1, Ocrl, Oit1, Olfm1, Olfm3, Olfml2b, Pamr1, Panx1, Pbxip1, Phlda1, Phyhipl, Pianp, Pld1, Plekhb1, Pls1, Pls3, Ppp1r17, Pygb, Ramp1, Rap2b, Rasgef1a, Rbm24, Rbms3, Rbp4, Rerg, Rgs20, Rhoc, Rtp4, S100a16, Scn3a, Scn3b, Sec14l4, Sema3e, Sema4a, Serpine2, Serping1, Sh3bgrl2, Slc16a7, Slc25a24, Slc2a3, Slc38a11, Slc4a4, Sst, Stk39, Ston2, Sult1d1, Syt5, Tagln3, Tenm4, Tiam1, Tmem130, Tmie, Tmtc1, Tox, Tox2, Tpm1, Trp53i11, Trpa1, Tspan12, Tspan8, Tst, Uchl1, Unc5c, Vat1l, Vim, Vopp1, Vsig2, Wfdc16, Zfp365.* | Baron et al. 2016 |
| δ_cells_3 | *Hhex, Rbp4, Sst.* | Baron et al. 2016 |
| ε_cells_31 | *Ghrl, Phgr1, Acsl1, Frzb, Ms4a8, Vtn, Apoh, Spink1, Vstm2l, Sptssb, S100a6, Fxyd3, Qdpr, F10, Clu, Krt18, Tmem176b, Gc, Rbp4, Pcsk1n, Tm4sf4, Krt8, Ttr, Anxa13, Amn, Tm4sf3, Cpm, Lgals4, Erbb3, Tmem45b, Hpn.* | Baron et al. 2016 |
| Endothelial | *Mcam, Vcam1, Pecam1, Sele, Cd34, Icam1, Itgb3, Eng, Cdh5, Tek, Kdr.* | Baron et al. 2016 |
| aStellate_13 | *Pdgfra, Col1a1, Fn1, Thy1, Lum, Mmp2, Timp1, Vcan, Negr1, Dcn, Lama2, Col5a2, Col6a3.* | Baron et al. 2016 |
| qStellate_11 | *Pdgfrb, Sparcl1, Gja4, Cspg4, Ednrb, Rgs5, Fabp4, Des, Itga1, Itga5, Mcam.* | Baron et al. 2016 |
| Acinar | *Ptprc, Prss1, Amy1, Cpa1, Ctrb1, Cela3a, Cela3b, Pla2g1b, Prss2, Clps, Pnlip, Sycn, Pnliprp1, Ctrc, Klk1, Cela2a, Cpb1, Ptf1a.* | Baron et al. 2016 |
| Schwann | *Sox10, S100b, Cryab, Ngfr, Plp1, Pmp22, Ngfr, Sox2, Foxd3, Id4, Gdnf, Nes, Scn7a.* | Baron et al. 2016 |
| Fibroblasts | *Mme, Itgb1, Cd47, Cd81, Lrp1, Il1r1.* | Baron et al. 2016 |
| Ductal | *Krt19, Tacstd2, Anxa2, S100a10, S100a11, Krt17, Krt18, Krt7, S100a16, S100a14, Tmp1.* | Baron et al. 2016 |
