# Supplementary material for: Pancreatic stellate cells have adipogenic and fibrogenic potentials but only show increased pro-fibrogenic propensity upon aging
Source: Redox Biol. 2025 Jul 29;86:103791. doi: 10.1016/j.redox.2025.103791 (PMC12337652; doi:10.1016/j.redox.2025.103791)
Supplement: Multimedia component 5 [file mmc5.docx]

**Materials and methods**

**Materials table**

| **Reagent or resource** | **Source** | **Identifier** |
| --- | --- | --- |
| **Antibodies** | | |
| Anti-Mouse CD31 (PECAM-1) FITC (Clone: 390) | Thermo Fisher Scientific | Cat# 11-0311-82  RRID:AB_465012 |
| Anti-Mouse CD45 FITC (Clone: 30-F11) | Thermo Fisher Scientific | Cat# 11-0451-82  RRID:AB_465050 |
| Anti-Mouse Ly-6A/E (Sca-1) APC (Clone: D7) | Thermo Fisher Scientific | Cat# 17-5981-82  RRID:AB_469487 |
| Anti-Mouse CD140a PE (Clone: APA5) | eBioscience | Cat# 12-1401-81  RRID:AB_657615 |
| Rabbit anti-PDGFRA Polyclonal Antibody | Invitrogen | Cat# PA5-16571  RRID:AB_10981626 |
| Goat anti-PDGFRα | R&D systems | Cat#AF1062; RRID:AB_2236897 |
| Alexa Fluor 488 goat anti-rabbit | Thermo Fisher Scientific | Cat#A-11008; RRID:AB_143165 |
| Alexa Fluor 568 goat anti-rabbit | Abcam | Cat#ab175471; RRID:AB_2576207 |
| Alexa Fluor 594 donkey anti-rabbit | Thermo Fisher Scientific | Cat#A-21207; RRID:AB_141637 |
| **Chemicals, Peptides, and Recombinant Proteins** | | |
| Collagenase Type II | Worthington | Cat#LS0004174 |
| Calcein Blue AM Viability Dye | eBioscience | Cat#65-0855-39 |
| Propidium Iodide (PI) | Sigma-Aldrich | Cat#P4170 |
| Roti-Histofix 4 % | Carl Roth | Cat#P087.3 |
| Triton™ X-100 | Sigma-Aldrich | Cat#X100 |
| Fluoromount-G | eBioscience | Cat#00-4958-02 |
| 4′,6-diamidino-2-phenylindole (DAPI) | BioLegend | Cat#422801 |
| Matrigel-Matrix | Corning | Cat#356231 |
| DMEM/high glucose | Pan Biotech | Cat#P04-03590 |
| Penicillin /Streptomycin | Pan Biotech | Cat#P06-07100 |
| Fetal Bovine Serum | Biochrom | Cat#S0115 |
| Basic fibroblast growth factor | Sigma-Aldrich | Cat#F0291 |
| DMEM/low glucose | Pan Biotech | Cat#P04-01550 |
| Insulin-transferrin-selenium (ITS) mix | Sigma-Aldrich | Cat#I3146 |
| RPMI-1640 | Pan Biotech | Cat#P04-18047 |
| Intercept® (TBS) Blocking Buffer | LI-COR Biosciences | Cat#927-60003 |
| TRIzol® Reagent | Fisher Scientific | Cat#12034977 |
| SeqAmp™ DNA Polymerase | Takara Bio, Inc. | Cat#: 638504 |
| Protector RNase Inhibitor | Sigma Aldrich | Cat#: 3335399001 |
| 1X TE Buffer | Thermo Fisher Scientific | Cat#: PN12090015 |
| SPRIselect | Beckman Coulter Life Sciences | Cat#: B23318 |
| Agencourt AMPure XP Beads | Beckman Coulter Life Sciences | Cat#: A63880 |
| **Software and algorithms used** | | |
| STAR (version 2.6.0a) | tabit.ucsd.edu/sdec | RRID:SCR_005622 |
| featureCounts (version 1.6.2) | bioinf.wehi.edu.au/featureCounts | RRID:SCR_012919 |
| Seurat (version 3) | satijalab.org/seurat/get_started.html | RRID:SCR_016341 |
| Cell Ranger (V4.0.0) | 10xgenomics.com | RRID:SCR_017344 |
| GraphPad Prism (version 9.4.1) | graphpad.com | RRID:SCR_002798 |
| GOplot | https://github.com/wencke/wencke.github.io | RRID:SCR_024419 |
| Cytoscape | cytoscape.org | RRID:SCR_003032 |
| ImageJ | imagej.net | RRID:SCR_003070 |
| Metascape | metascape.org | RRID:SCR_016620 |
| CFX Manager | Bio-Rad | RRID:SCR_017251 |
| FlowJo™ v10.5.3 | BD Biosciences | RRID:SCR_008520 |
| **Critical Commercial Assays** | | |
| RNA Miniprep Kit | Zymo Research | Cat#1065 |
| High Capacity cDNA Reverse Transcription Kit | Fisher Scientific | Cat#10186954 |
| Maxima SYBR Green/ROX qPCR Master Mix | Fisher Scientific | Cat#11893913 |
| Agilent High Sensitivity DNA Kit | Agilent Technologies | Cat#: 5067- 4626 |
| Agilent DNA 1000 Kit | Agilent Technologies | Cat#: 5067-1504 |
| Chromium Next GEM Single Cell 3' Kit v3.1, 4 rxns | 10X Genomics | Cat#: PN1000269 |
| Chromium Next GEM Chip G Single Cell Kit | 10X Genomics | Cat#: PN-1000127 |
| Agilent High Sensitivity DNA Kit | Agilent Technologies | Cat#: 5067- 4626 |
| **Deposited Data** | | |
| scRNA-seq 10x-data? |  |  |
| **Experimental Models: Organisms/Strains** | | |
| Mouse, strain: C57BL/6J | The Jackson Laboratory | RRID:IMSR_JAX:000664 |
| B6.129S4-Pdgfratm11(EGFP)Sor/J | The Jackson Laboratory | RRID:IMSR_JAX:007669 |
| **Oligonucleotides** | | |
| Fwd: GTTCTCAGCCCAACAATACAAGA  Rev: GTGGACGGGTCGATGTCAC | This manuscript | Nos2: mouse, NM_ 010927.3 |
| Fwd: GCCTGAGTGGCTGTCTTTTGA  Rev: CACAAGAGCAGTGAGCGCTGAA | This manuscript | Tgfb1: mouse, NM_011577 |
| Fwd: CGAGGCGAGATTTGCAGGTATT  Rev: TTAGCAGGAGATGTGGGGTCTT | This manuscript | Tgfb2: mouse,  NM_009367.4 |
| Fwd: TGACCTGGAGGAAAACATTAAGA  Rev: AGCCCTGTATGTCTTCACACTG | This manuscript | Ctgf: mouse,  NM_010217.1 |
| Fwd: TGTGTTCCCATCCATCGTGG  Rev: GATGTCGCGCACAATCTCAC | This manuscript | Acta1: mouse, NM_009606.1 |
| Fwd: GTGCTCCTGGTATTGCTGGT  Rev: GGCTCCTCGTTTTCCTTCTT | This manuscript | Col1a1: mouse,  NM_007742 |
| Fwd: ACGTAAGCACTGGTGGACAGA  Rev: GAGGGCCATAGCTGAACTGA | This manuscript | Col3a1: mouse,  NM_009930.2 |
| Fwd: GACCTGCAGTGGACTTACCC  Rev: GGCAGCACATTCATACTCTCC | This manuscript | Pdgfra: mouse, XM_006504263.5 |
| Fwd: ATACCCCGGGAGTTGATCGA  Rev: TCTGTCTCCTCCTCCCGATG | This manuscript | Pdgfa: mouse, NM_001422139.1 |
| Fwd: CGGAGCTCAGTGAGAGGAAG  Rev: CGGAGTCCATAGGGAGGAAG | This manuscript | Pdgfrb: mouse,  XM_030250361.2 |
| Fwd: AACGATGATGCACTTGCAGA  Rev: TGGTACTCCAGAAGACCAGAGG | This manuscript | Il6: mouse,  NM_031168.2 |
| Fwd: CTCCAAGCCAAAGTCCTTAGAG  Rev: AGGAGCTGTCATTAGGGACATC | This manuscript | Arg1: mouse, NM_007482.3 |
| Fwd: CCCGATGGCGAGATCATGAA  Rev: TCGCTAGAGCAGGTCTGTCT | This manuscript | Ccn2: mouse,  NM_ 010548.2 |
