# Supplementary figures and images for: Pancreatic stellate cells have adipogenic and fibrogenic potentials but only show increased pro-fibrogenic propensity upon aging
Source: Redox Biol. 2025 Jul 29;86:103791. doi: 10.1016/j.redox.2025.103791 (PMC12337652; doi:10.1016/j.redox.2025.103791)

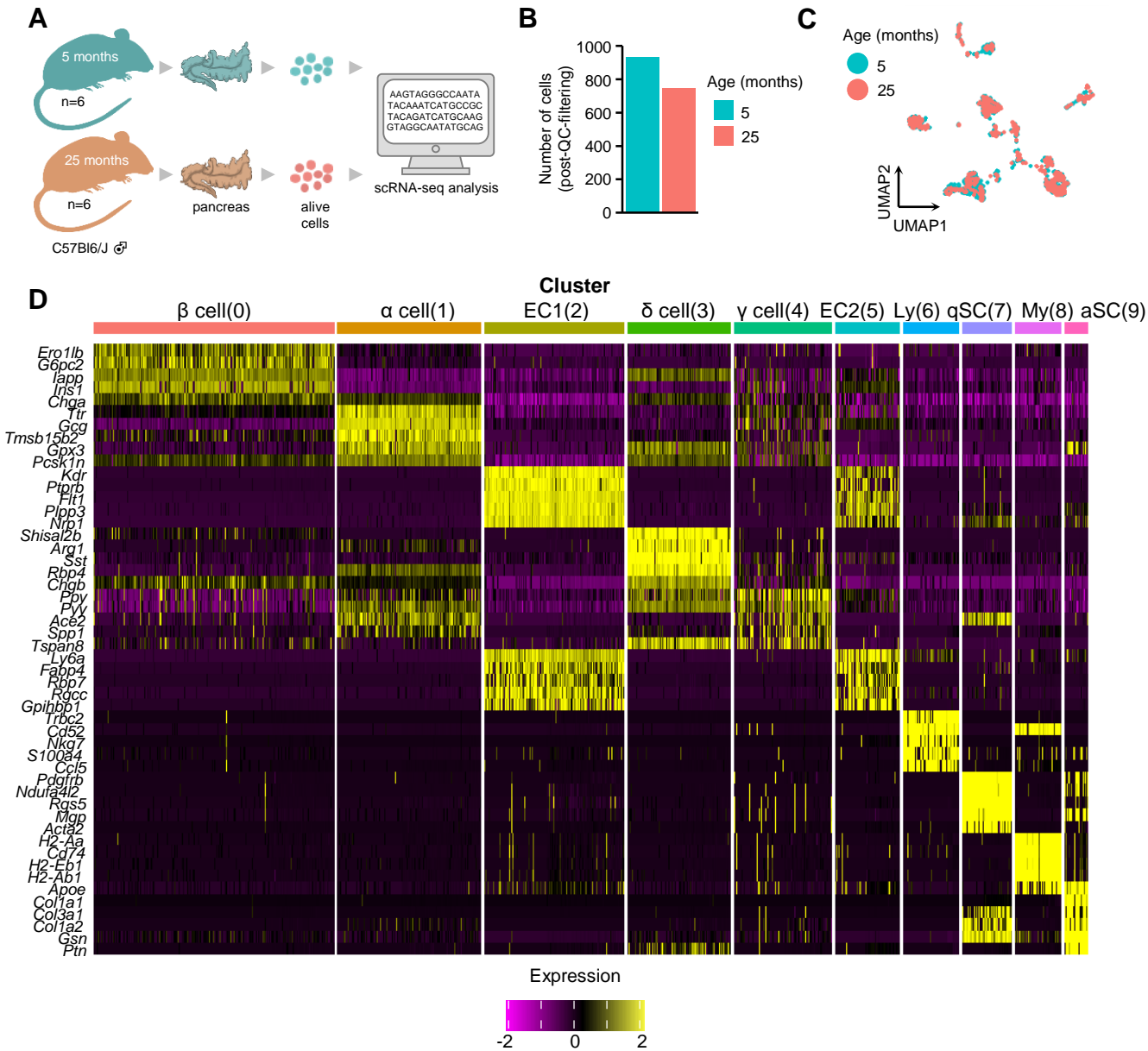

## Supplementary Figure S2

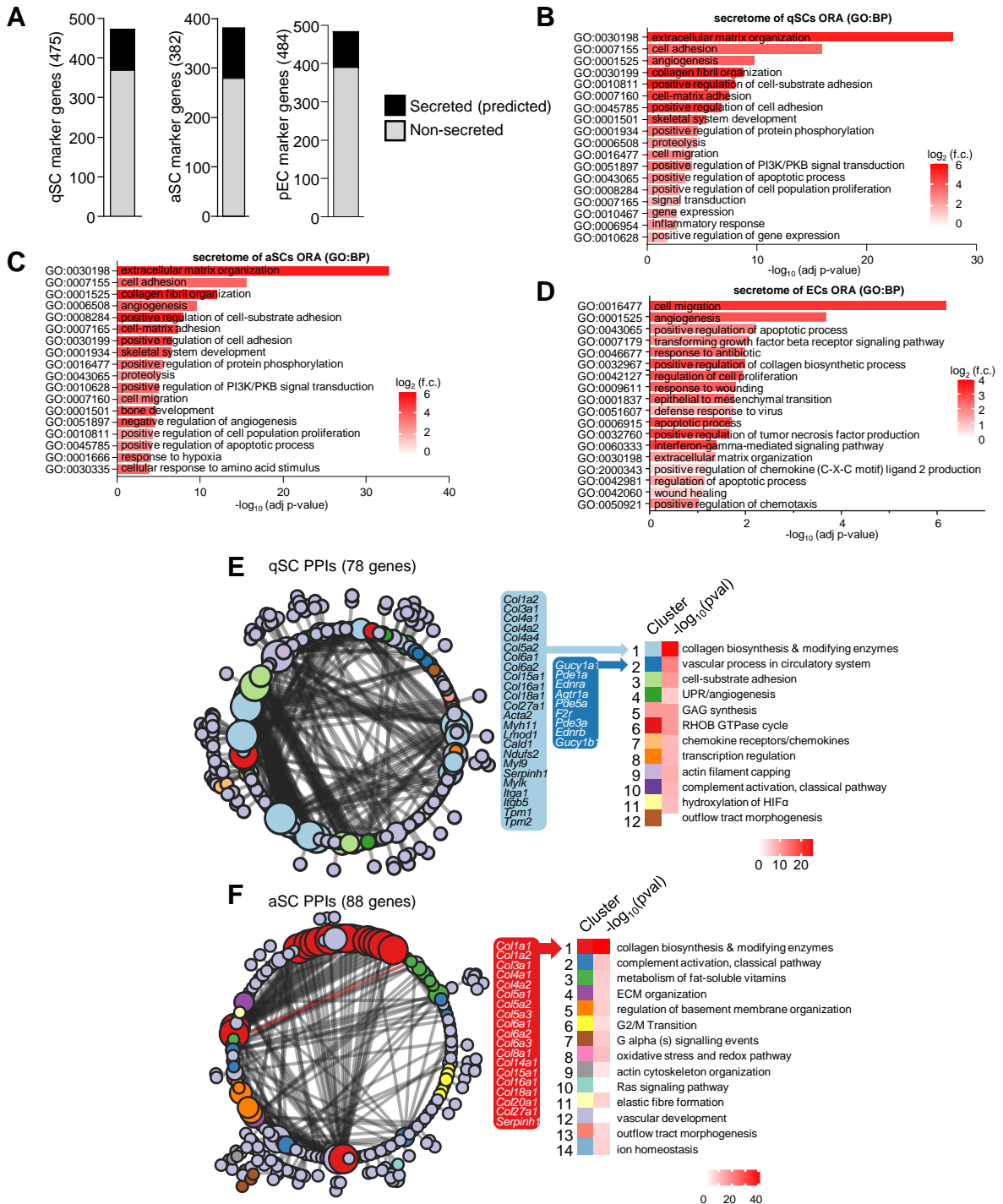

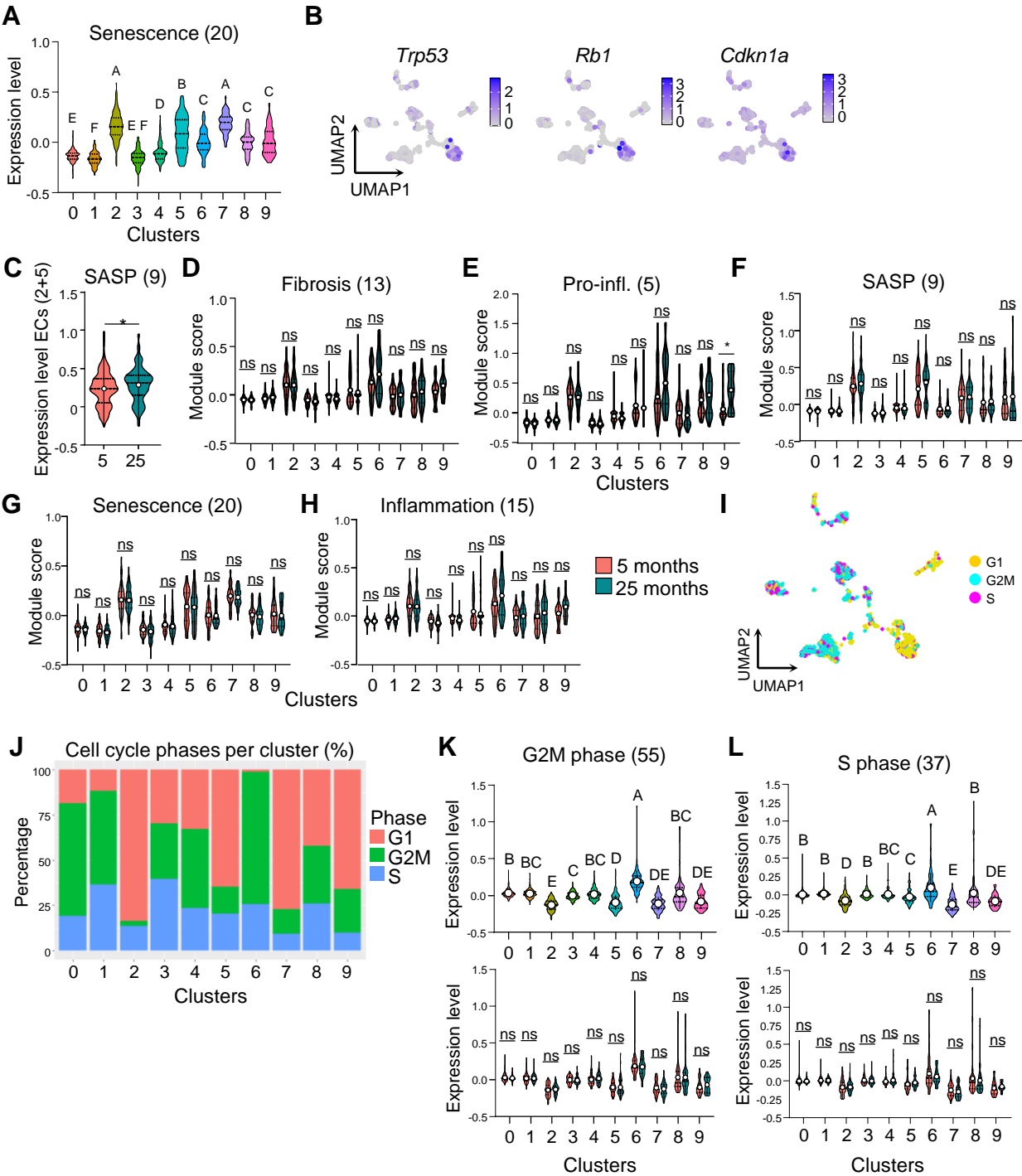

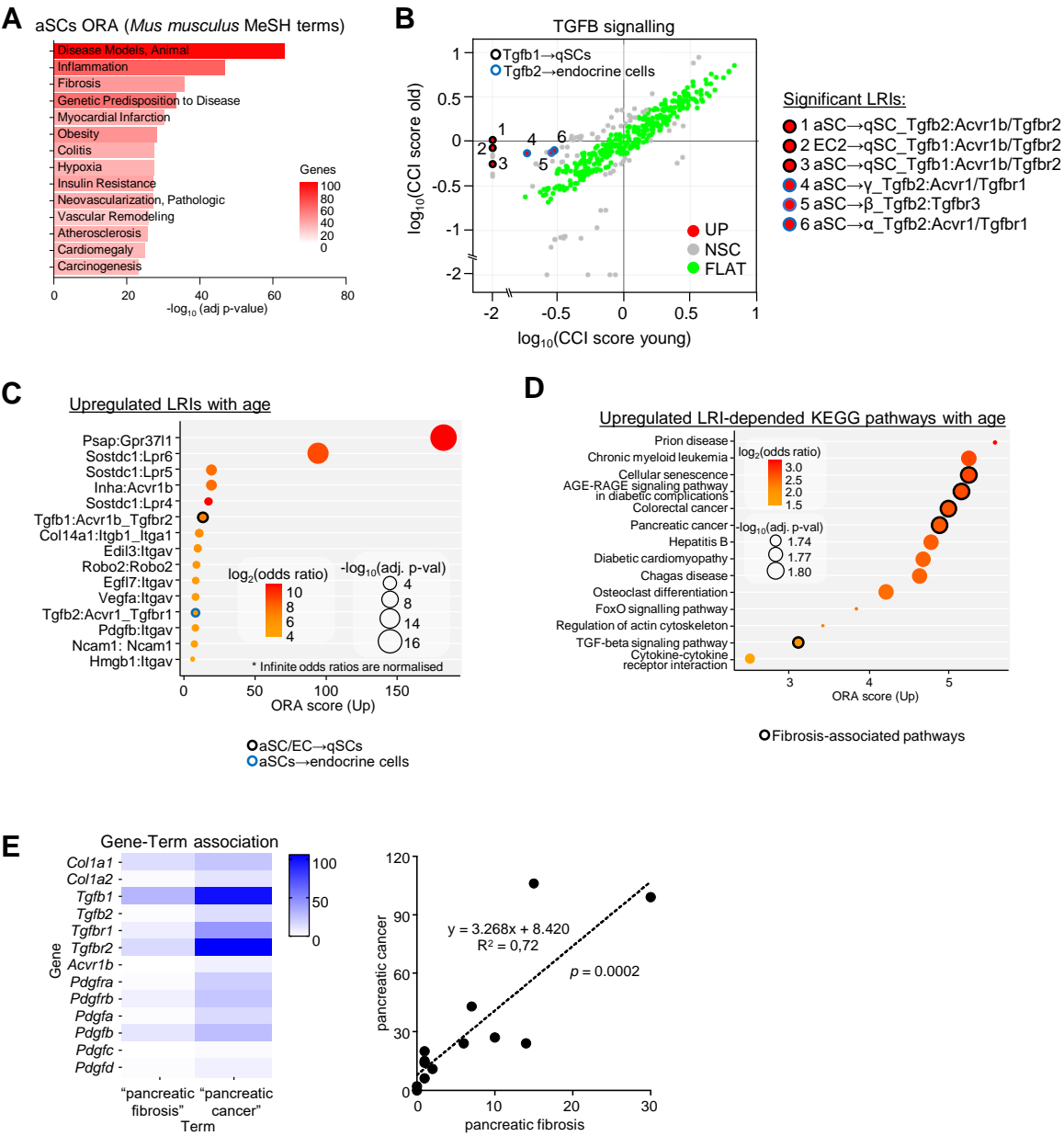

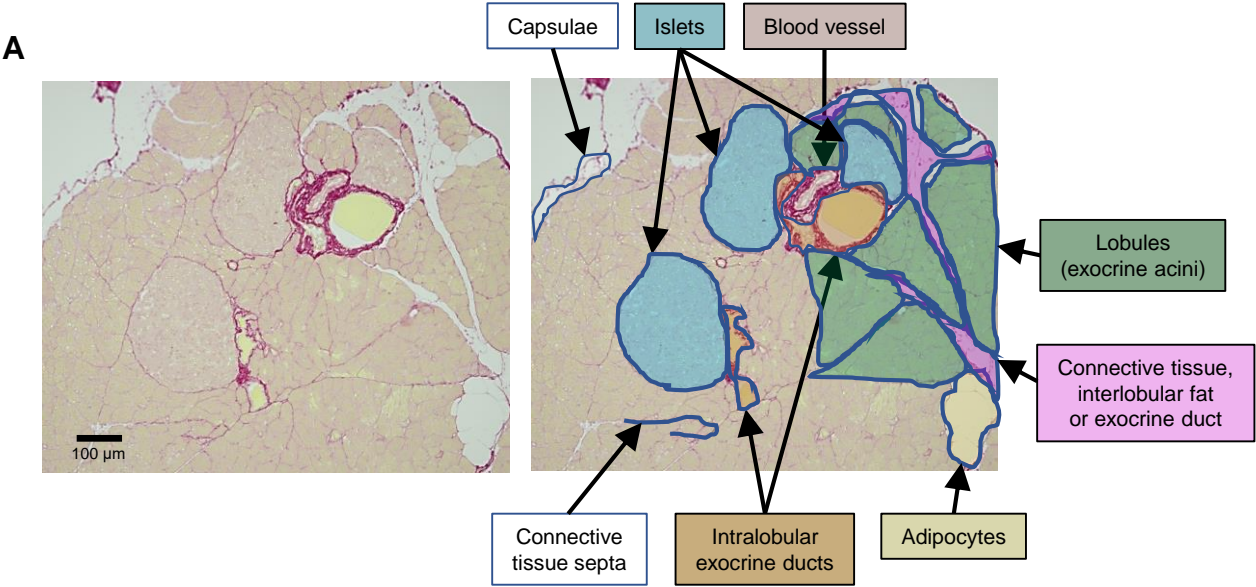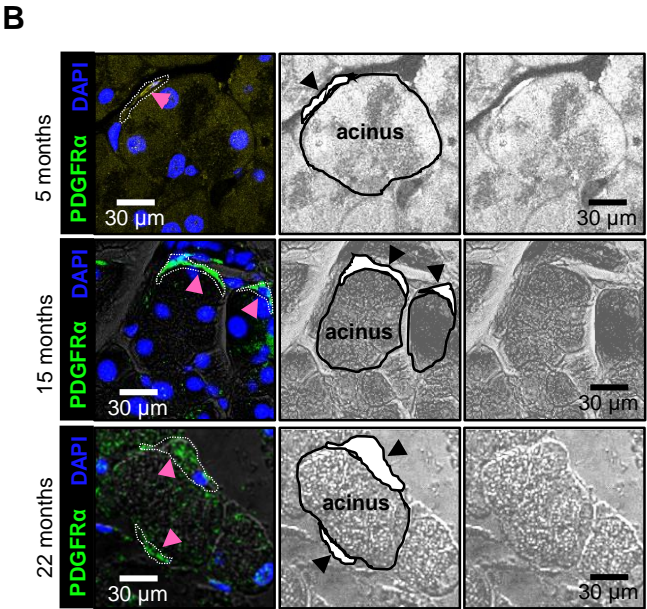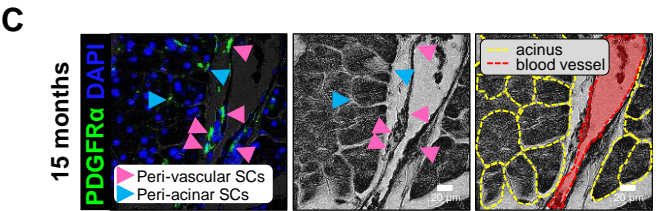

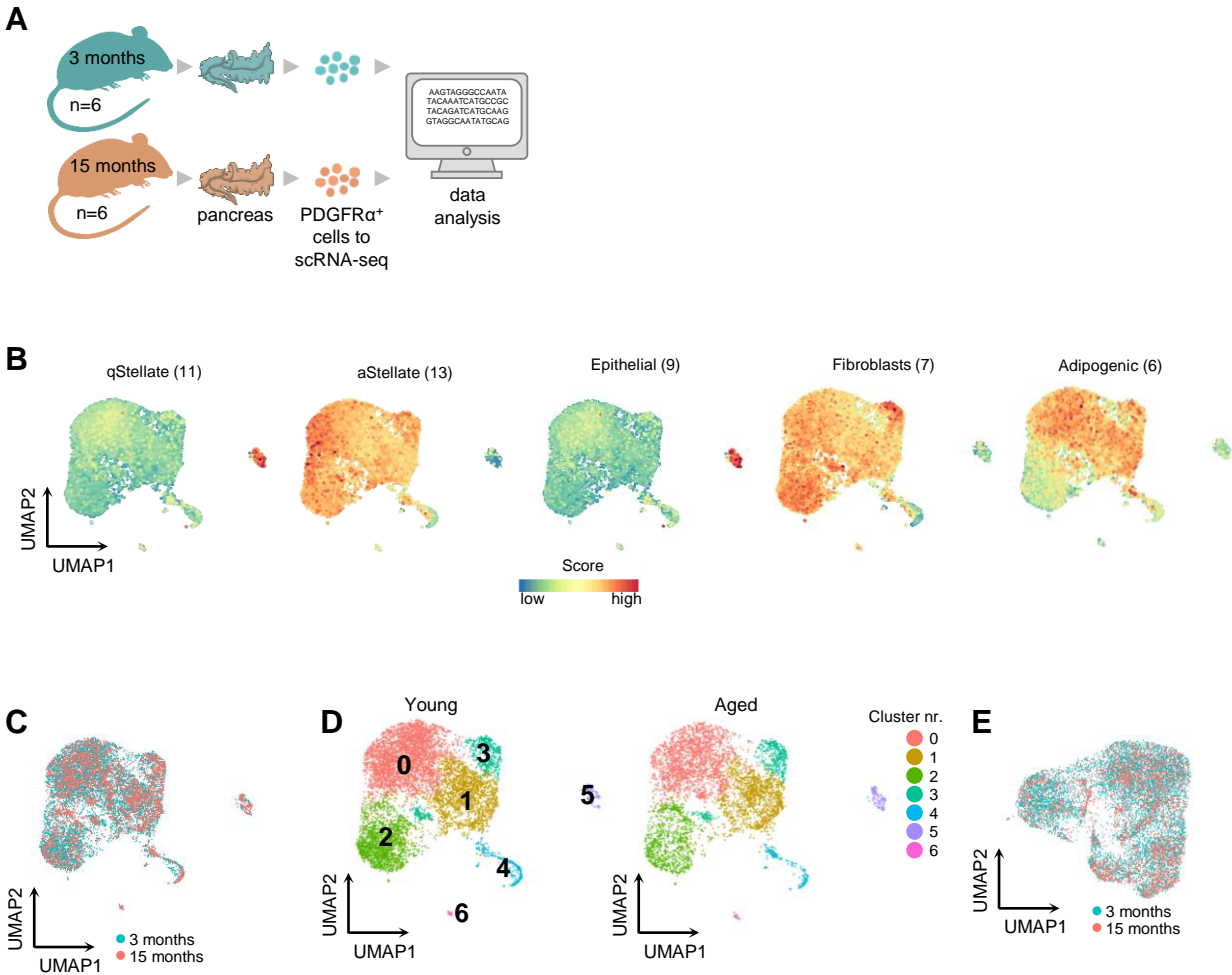

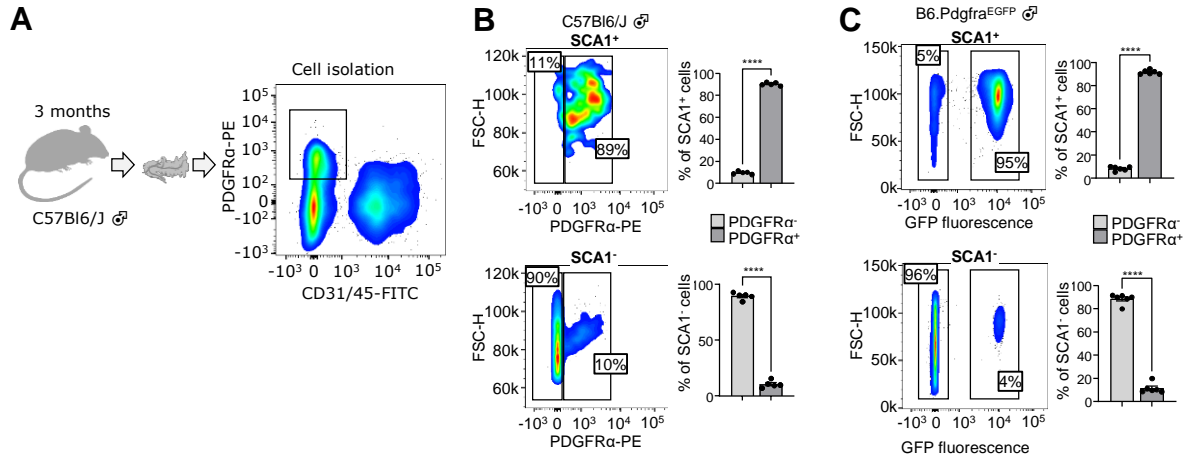

Supplement: Multimedia component 7 — Supplementary Figure S1 (related to main Fig. 1) Overview of heterogenous pancreatic cell type scRNA-seq analysis and of distinct transcriptomes in heterogeneous cell subsets. (A) Overview of experimental design. Diverse cell populations were isolated from adult (5 months) and very aged (25 months) male C57Bl/6J mice for the analyses shown. To also capture endocrine cell heterogeneity, samples were partially enriched for islets, and alive cells were sorted for scRNA-seq analysis. (B) Cell numbers in scRNA-seq data. Bar chart showing the total number of cells for the two age groups. (C) Representation of cell populations across age groups. scRNA-seq data of 1680 pancreas resident heterogeneous cells visualized by UMAP dimensionality reduction and age group, belonging to adult (5 months) or very aged (25 months) mice. (D) Heatmap-visualization of the top 5 marker genes representing cluster-specific DEGs (Wilcoxon Rank sum test, p < 0.01). Supplementary Figure S2 (related to main Fig. 2, Fig. 3) aSCs express and secrete most collagen type I (Col1a1). (A) Secretome analysis of quiescent stellate cells (qSCs), activated stellate cells (aSCs), and endothelial cells (ECs). VerSeDa in silico analysis showing the predicted secreted (black bars) and predicted non-secreted (grey bars) gene products for the respective cell type-specific marker genes representing cluster-specific DEGs (Wilcoxon Rank sum test, p < 0.01). (B) In silico predicted secretome over-representation GO:BP analysis for qSCs, showing terms in decreasing order of significance (-log10 p-value, x axis) identifying extracellular matrix (ECM) and angiogenic processes. (C) In silico predicted secretome over-representation GO:BP analysis for aSCs, showing terms in decreasing order of significance (-log10 p-value, x axis) identifying extracellular matrix (ECM) and collagen processes. (D) In silico predicted secretome over-representation GO:BP analysis for ECs, showing terms in decreasing order of significance (-log [file mmc7.pdf]
